# Root-TRAPR: a modular plant growth device to visualize root development and monitor growth parameters, as applied to an elicitor response of *Cannabis sativa*

**DOI:** 10.1186/s13007-022-00875-1

**Published:** 2022-04-09

**Authors:** Pipob Suwanchaikasem, Alexander Idnurm, Jamie Selby-Pham, Robert Walker, Berin A. Boughton

**Affiliations:** 1 School of BioSciences, University of Melbourne, Melbourne, VIC 3010 Australia; 2 Nutrifield Pty Ltd, Melbourne, VIC 3020 Australia; 3 Australian National Phenome Centre, Murdoch University, Perth, WA 6150 Australia

**Keywords:** 3D printing, Chitinase, Chitosan, EcoFAB, Exudate, Hydroponic, Industrial hemp, Peroxidase, Plant defense, Phytohormone

## Abstract

**Background:**

Plant growth devices, for example, rhizoponics, rhizoboxes, and ecosystem fabrication (EcoFAB), have been developed to facilitate studies of plant root morphology and plant-microbe interactions in controlled laboratory settings. However, several of these designs are suitable only for studying small model plants such as *Arabidopsis thaliana* and *Brachypodium distachyon* and therefore require modification to be extended to larger plant species like crop plants. In addition, specific tools and technical skills needed for fabricating these devices may not be available to researchers. Hence, this study aimed to establish an alternative protocol to generate a larger, modular and reusable plant growth device based on different available resources.

**Results:**

Root-TRAPR (Root-Transparent, Reusable, Affordable three-dimensional Printed Rhizo-hydroponic) system was successfully developed. It consists of two main parts, an internal root growth chamber and an external structural frame. The internal root growth chamber comprises a polydimethylsiloxane (PDMS) gasket, microscope slide and acrylic sheet, while the external frame is printed from a three-dimensional (3D) printer and secured with nylon screws. To test the efficiency and applicability of the system, industrial hemp (*Cannabis sativa*) was grown with or without exposure to chitosan, a well-known plant elicitor used for stimulating plant defense. Plant root morphology was detected in the system, and plant tissues were easily collected and processed to examine plant biological responses. Upon chitosan treatment, chitinase and peroxidase activities increased in root tissues (1.7- and 2.3-fold, respectively) and exudates (7.2- and 21.6-fold, respectively). In addition, root to shoot ratio of phytohormone contents were increased in response to chitosan. Within 2 weeks of observation, hemp plants exhibited dwarf growth in the Root-TRAPR system, easing plant handling and allowing increased replication under limited growing space.

**Conclusion:**

The Root-TRAPR system facilitates the exploration of root morphology and root exudate of *C. sativa* under controlled conditions and at a smaller scale. The device is easy to fabricate and applicable for investigating plant responses toward elicitor challenge. In addition, this fabrication protocol is adaptable to study other plants and can be applied to investigate plant physiology in different biological contexts, such as plant responses against biotic and abiotic stresses.

**Supplementary Information:**

The online version contains supplementary material available at 10.1186/s13007-022-00875-1.

## Background

In nature, plant roots develop underground and in sophisticated associations with microorganisms, making it challenging to observe root structure and conduct research on root activities [1]. Therefore, several platforms, for example, rhizotrons [2], rhizoponics [3] and rhizoboxes [4], have been developed to facilitate plant root morphological studies in controlled laboratory settings. In addition, technologies like Plant-in-Chip [5], RootChip [6], tracking roots interaction system (TRIS) [7], and ecosystem fabrication (EcoFAB) [8] have been further modified to increase the accessibility of plant-microbe interaction analysis. However, these systems are custom-made, requiring specialized techniques, tools and settings for manufacturing and implementation. Therefore, modification of the designs may be necessary upon the availability of different resources and intended research application.

One of the most recent examples, EcoFAB (https://eco-fab.org/), is an inexpensive and easy-to-fabricate device built based on three-dimensional (3D) printing technology [9, 10]. The original iteration is constructed using a microscope glass slide bonded via a plasma cleaner to custom-built polydimethylsiloxane (PDMS) growth chamber. The PDMS section is cast in a plastic mold, printed from a 3D printer. Optionally, an attachment between the glass slide and the PDMS layer can be reversibly bound using a 3D printed plastic or a machined metal clamp. The EcoFAB model has many benefits. It enables readily accessible observation of root morphology and microbial localization using microscopes and other non-destructive imaging tools. Root biochemical and exudate composition can be collected and analyzed under standardized procedures. The model can use different growth substrates such as soil, sand and liquid [9]. The reproducibility of the EcoFAB device has been verified across multiple laboratories in diverse growth environments [11]. The versatility of the EcoFAB system permits robust studies on model plants such as *Arabidopsis thaliana*, *Brachypodium distachyon* and *Panicum virgatum*. Although appropriate for these model plants, a larger device is required to address research questions in a broader array of plant species like staple and industrial crops, which are generally longer lived and grow to larger sizes than the model plants. Moreover, technical support, including 3D printers, plastic materials and accompanying tools, may vary across different workplaces. Hence, manufacturing processes are dependent upon the availability of the relevant machinery and supplies.

Industrial hemp (*Cannabis sativa*) is an emerging crop within the agricultural industry worldwide [12]. Its global market is projected to increase from $3.5 billion in 2019 to $18.8 billion in 2025, with a compound annual growth rate of 32.17% [13]. Hemp seed contains low tetrahydrocannabinol (THC) content but a high amount of protein and a good proportion of healthy unsaturated fatty acids [14, 15], creating the demand in the food and beverage industries. Hemp seed oil is a nutritional supplement added to skincare and medicinal products [12, 16]. In addition, hemp fiber is a perfect source for the textile industry owing to its robustness, and high absorbent capacity [17] and hemp hurd has been increasingly processed into hempcrete to replace traditional concrete in construction and building [18].

Despite its benefits, fundamental research to inform and establish daily agronomical practice has been lacking and inconclusive for the growers, who have been unable to transform scientific data into field applications [19]. For example, *C. sativa* is infected by several pathogenic fungi such as *Botrytis cinerea,* causing grey mold, *Fusarium* and *Pythium* species causing root rot, *Macrophomina phaseolina* causing charcoal rot, *Sclerotinia sclerotiorum* causing stem canker and *Golovinomyces cichoracearum* causing powdery mildew [20–22]. These infections suppress plant growth and reduce yield and product quality in outdoor fields and greenhouse settings [21]. However, the pathology underlying the different infections is poorly understood, and disease management programs have not been fully established [22]. The growers may apply inorganic agents, for example, potassium bicarbonate, hydrogen peroxide, boric acid, orthosilicic acid or synthetic fungicides such as fluopyram, to moderate or eradicate fungal pathogens [23]. To avoid using chemicals, natural products such as seaweed extract, plant growth-promoting bacteria, humic substances, and chitin/chitosan derivatives have been used to increase product yield and promote plant defense to combat pests and diseases in other crops [24, 25]. They can be mixed into the soil or diluted and sprayed on aboveground plant tissues [26, 27]. Nonetheless, the benefits of any approach have not yet been comprehensively examined in *C. sativa* plants. Verifying their stimulating effects will benefit both industrial hemp and medicinal cannabis (high-THC cultivars) industries to tackle fungal disease problems in the field.

As principally inspired by the EcoFAB model, we developed a new device called Root-Transparent, Reusable, Affordable 3D Printed Rhizo-hydroponic or Root-TRAPR system. The device was enlarged and strengthened to cope with industrial hemp growth. To demonstrate the usability and effectiveness of the system, an elicitor challenge assay using colloidal chitosan was developed. Its effect was examined on plant defense by monitoring plant root development and analyzing biological responses by measuring specific enzymatic activities and phytohormone levels. The Root-TRAPR system could be a convenient testing platform for verifying the stimulating effects of plant elicitors on *C. sativa* plants to further the goals of sustaining and promoting the expanding cannabis industry.

## Results

### Generation of Root-TRAPR system

**Fig. 1:**
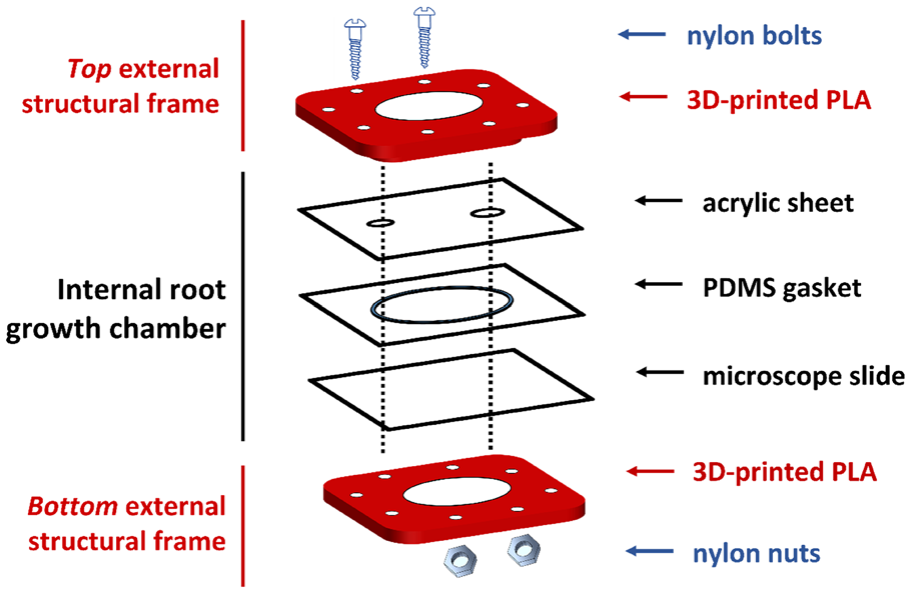
Exploded-view diagram displaying the main components of the Root-TRAPR system. The internal root growth chamber comprises an upper acrylic sheet, a customized PDMS gasket and a bottom microscope slide. The external structural frames (top and bottom) are made of 3D-printed PLA plastic, retained with nylon bolts and nuts (× 8). All components are stable to ethanol, facilitating decontamination and sterilization

**Fig. 2:**
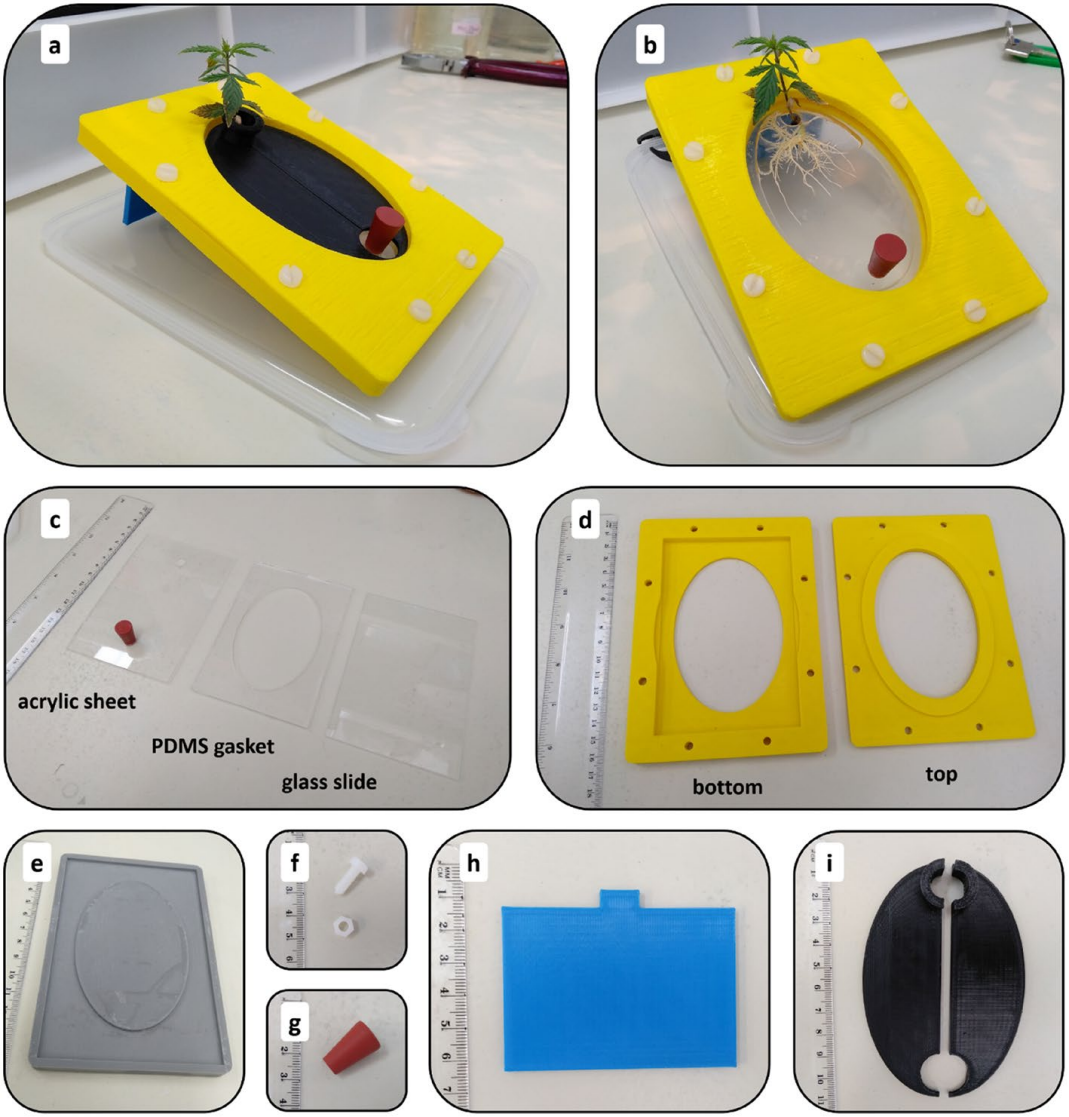
Pictures displaying a complete assembled Root-TRAPR system with industrial hemp grown inside (**a** and **b**) and all individual parts (**c**–**i**) – the internal root growth chamber consisted of an acrylic sheet, a PDMS gasket and a microscope slide (**c**), top and bottom external structural frames (**d**), PDMS mold (**e**), nylon bolts and nuts (**f**), rubber bung (**g**), stand (**h**) and window shutter (**i**)

Through a process of iterative design, the Root-TRAPR system was created based on available resources at the University of Melbourne, Australia. The model was inspired by a range of plant growth devices, including the recent EcoFAB model developed at Lawrence Berkeley National Laboratory, US [9]. We retain some elements of the original EcoFAB design, including a glass microscope slide base with a PDMS layer. Differently, our chamber is enclosed by an acrylic sheet, sealed using a compression seal supported by a 3D-printed external structural frame. An exploded-view diagram displaying the components of the Root-TRAPR system and the assembly is shown in Fig. 1. Representatives of the system and all components are featured in Fig. 2, and the details of each part are described in Table 1.

**Table 1:** Details and cost of each component in the Root-TRAPR system

No	Component	Material	Size	Approximate cost (AU$) per unit	Supplier
1	PDMS mold	PLA	External: 138 × 94 mm Internal: 130 × 86 mm	$9.0	Printed from a 3D printer
2	PDMS layer (Void oval chamber)	PDMS (Sylgard 184)	130 × 86 mm; 3 mm (100 × 64 mm)	$6.5	Cast in the PDMS mold
3	Microscope slide	Glass	128 × 85 mm; 1 mm	$2.5	ProSciTech, Australia
4	Acrylic sheet (Small hole) (Large hole)	Transparent acrylic plastic	128 × 85 mm; 1.5 mm (8 mm in diameter) (9 mm in diameter)	$2.0	Warlond Plastics, Australia
5	Rubber bung	Rubber	8 × 13 mm; 19 mm	$0.5	Pacific Laboratory Products, Australia
6	Top frame	PLA	External: 162 × 119 mm; 3.5 mm Internal (oval): 130 × 90 mm; 4 mm	$15.0	Printed from a 3D printer
7	Bottom frame	PLA	External: 162 × 119 mm; 9 mm Internal: 132 × 88 mm; 6.5 mm	$19.0	Printed from a 3D printer
8	Cheese head bolts (8 pieces)	Nylon	5 × 40 mm	$2.5	Keables, Australia
9	Hexagon nuts (8 pieces)	Nylon	5 mm	$1.0	Keables, Australia
10	Window shutter	PLA	106 × 70 mm; 2 mm	$4.0	Printed from a 3D printer
11	Stand	PLA	72 × 50 mm; 3 mm	$2.0	Printed from a 3D printer
			Total	$64.0	

The Root-TRAPR system comprises of two major components—an internal root growth chamber (Fig. 2c) and an external structural frame (Fig. 2d). The internal root growth chamber has a transparent viewing configuration from either top or bottom sides through a transparent acrylic sheet and microscope slide, respectively, to facilitate plant root structure observation. A square PDMS gasket with an oval void in the center is pre-cast in a 3D-printed plastic mold (Fig. 2e), enabling fine tuning the void volume by increasing/decreasing the oval width or gasket thickness. The acrylic sheet is pre-drilled with upper and lower holes to insert the plant seed and exchange plant growing media. The elastic PDMS gasket is inserted between the acrylic sheet and microscope slide to create a root growth chamber.

The three internal layers comprised of acrylic sheet, PDMS gasket and microscope slide are secured and compressed using top and bottom external frames printed from a fused deposition modeling (FDM) 3D printer using an inexpensive polylactic acid (PLA) plastic material. The frame is furnished with eight sets of nylon bolts and hexagon nuts (Fig. 2f) to fasten and compress the whole model together tightly. A rubber bung (Fig. 2g) is plugged into the lower smaller hole of the acrylic sheet to stop leakage. During growth experiments, the stand (Fig. 2h) and window shutter (Fig. 2i) can be additionally put in place to tilt the model at a 25° angle to promote gravitropism and prevent direct light onto the plant roots, respectively. The assembled Root-TRAPR device is not damaged by absolute ethanol, therefore the model can be submerged in the solvent for decontamination and sterilization before use.

The approximate cost of the Root-TRAPR system is detailed in Table 1. All 3D-printed objects are subjected to a subsidized AU$0.15 per 1 g material according to the standard printing price for the University of Melbourne [28]. The total cost is approximately AU$64.0 per unit but could vary based on differing plastic materials, printing resolution, machinery techniques or bulk supplies used.

### Industrial hemp growth in Root-TRAPR system

Plant growth experiments were carried out using three biological replicates under two different condition—control and chitosan treatment. After germination in Petri dishes, industrial hemp seedlings were transferred to the Root-TRAPR systems and maintained for 14 days in a controlled environment with Hoagland nutrient solution (Additional file 1). After 7 days of growth, nutrient solutions were exchanged. Control plants were treated with standard Hoagland solution and chitosan treatment was performed by substituting plain Hoagland solution with the solution containing 1% w/v colloidal chitosan. Plant growth was monitored with root structure recorded every 2–3 days using a modified scanner connected with the WinRHIZO software. Upon harvest on day 14, plant root and shoot tissues and root exudate were collected and subsequently processed for enzymatic assays, phytohormone quantifications and gene detections.

**Fig. 3:**
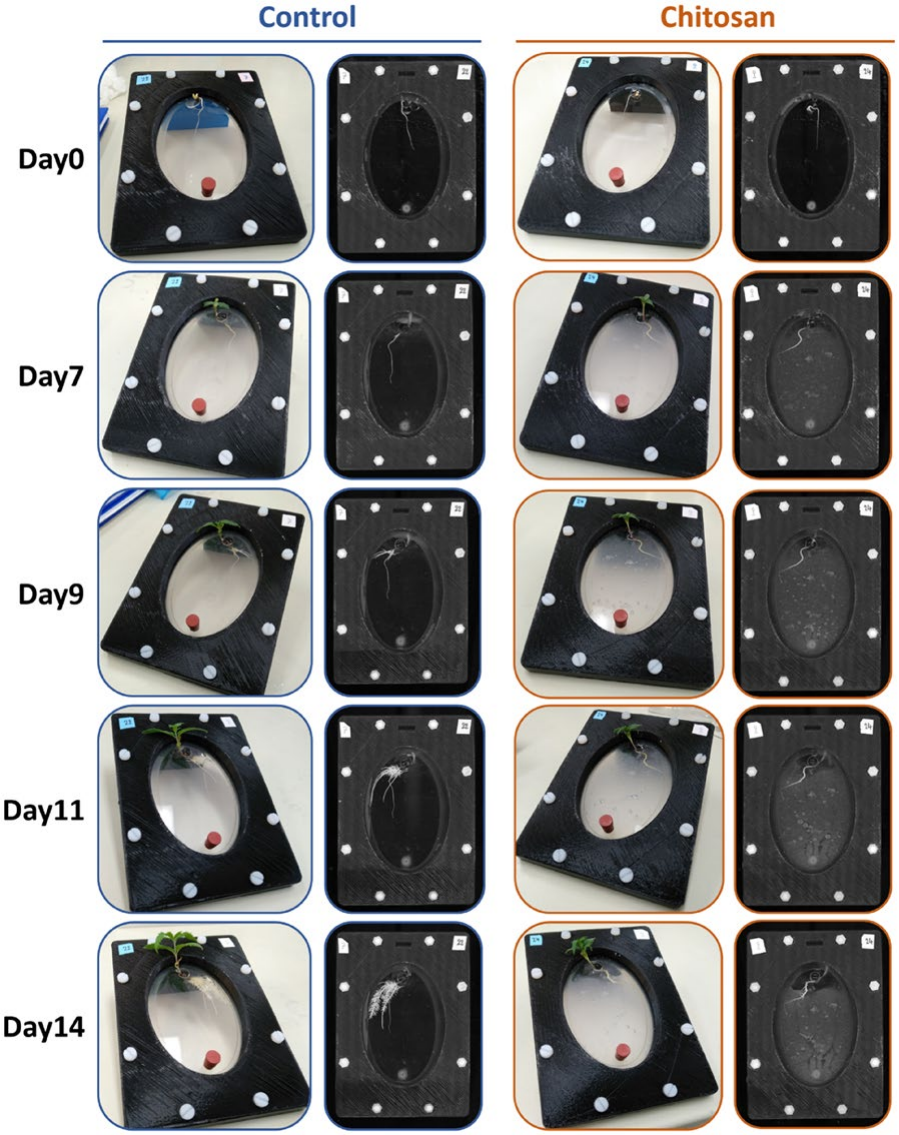
Photos presented sequential development of industrial hemp in the Root-TRAPR system comparing control and chitosan-treated condition from day 0 to day 14. Chitosan treatment was conducted on day 7 of the experiment. Left-panel pictures of each condition show top-view photos taken on a smartphone camera. Right-panel pictures represent root images captured using a WinRHIZO root scanner

**Fig. 4:**
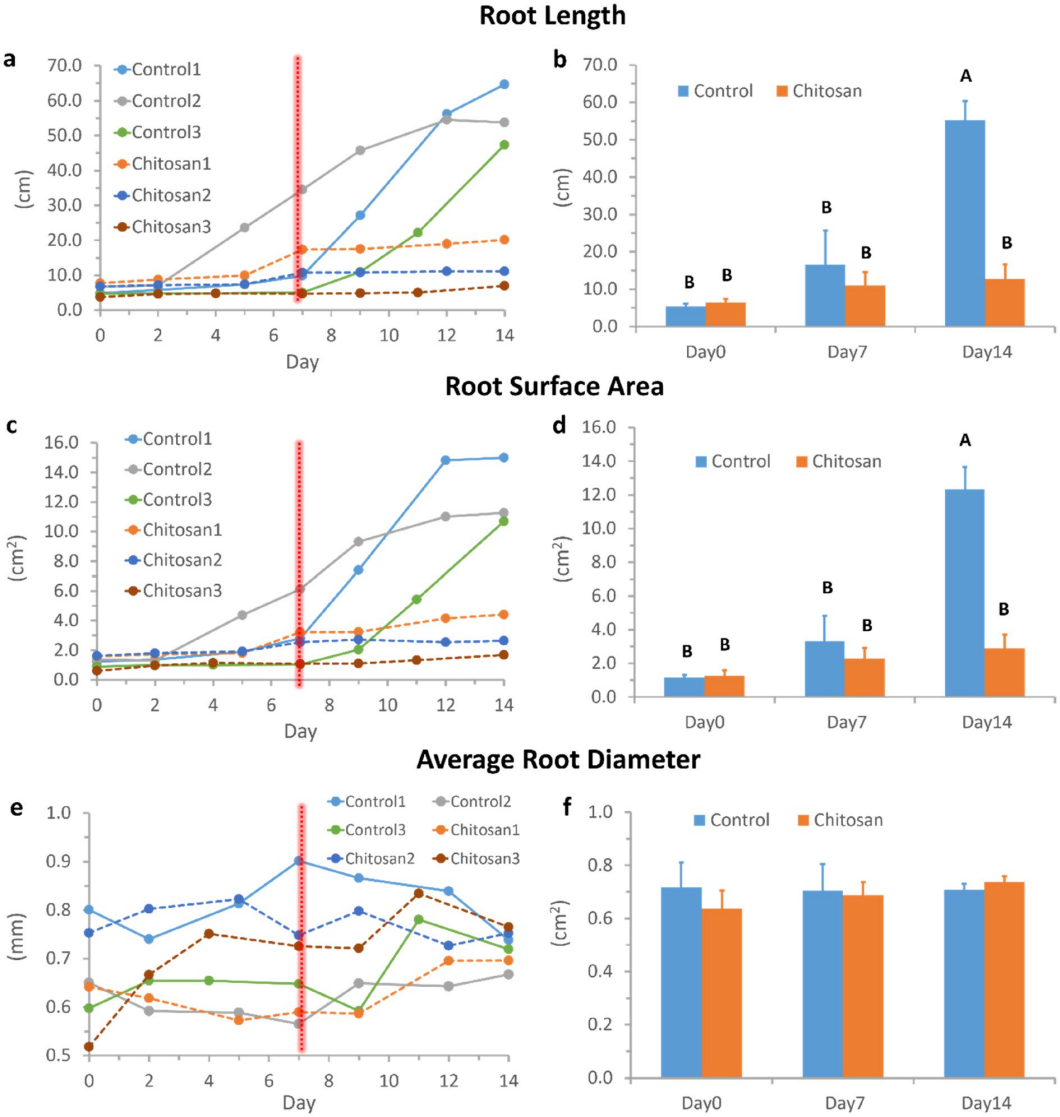
Graphs depicting root developments of industrial hemp in the Root-TRAPR system comparing control and chitosan-treated plants. The measurements are root length (**a** and **b**), root surface area (**c** and **d**), and average root diameter (**e** and **f**). Line graphs (**a**, **c** and **e**) display the root development of three individual replicates of each group. Bar graphs (**b**, **d** and **f**) show an average with an error bar of the standard error of the mean (SEM). Different capital letters above bar graph (A and B) refer to a significant difference at p-value < 0.05 tested by one-way ANOVA, followed by Tukey's HSD test across treatment and day

Root morphology was captured by a well-calibrated optical light scanner and analyzed by the WinRHIZO software throughout the study (Fig. 3). Root growth was monitored through three different parameters—root length, root surface area and average root diameter. Under control conditions, plants constantly expanded their roots throughout 14 days of observation, ending at 55.27 ± 5.06 cm and 12.33 ± 1.35 cm^2^ in length and surface area, respectively (Fig. 4a–d). The expansion rate was slow during the first week (from 5.47 to 16.49 cm in length and 1.17 to 3.32 cm^2^ in surface area) but increased during the second week (from 16.49 to 55.27 cm in length and 3.32 to 12.33 cm^2^ in area). Despite enlarging in root length and surface area, the average root diameter did not change during the monitoring period (0.72 to 0.71 mm; Fig. 4e–f). This indicates that plants expanded existing roots to a larger size and at the same time generated new lateral roots. Young secondary and tertiary branch roots, ranging between 0.2–0.5 mm in diameter, offset the larger primary and pre-existing branch roots (Fig. 3). Therefore, average root diameter of the control plants remained constant.

In the chitosan-treated group, plant roots developed well before chitosan was introduced on day 7, cumulative at 10.98 ± 3.65 cm in total root length and 2.28 ± 0.63 cm^2^ in root surface area, which were not significantly different from the controls (Fig. 4a–d). However, after the treatment, plants displayed significantly reduced root expansion, finishing at 12.79 cm ± 3.89 in length and 2.91 ± 0.80 cm^2^ in surface area, which were significantly smaller than those of control plants (p-value = 0.003 and 0.004, respectively). The average root diameter was slightly increased after the treatment, expanding from 0.69 ± 0.05 mm (day 7) to 0.74 ± 0.02 mm (day 14) but was not statistically significant and did not differ from the controls (Fig. 4e–f). As observed from the root morphology (Fig. 3), chitosan-treated plants generated remarkably fewer new branch roots than the controls. This could reflect the slight increase in root diameter of chitosan-treated plants.

The reduction of root expansion after exposure to chitosan is consistent with previous observations on *Arabidopsis thaliana* [29, 30]. In those studies, plants struggled to elongate roots when exposed to chitosan from as low as 0.01% w/v in concentration. The chitosan disruption on root growth is unlikely to be caused by an increase in viscosity or osmolality of the chitosan suspension. In preparation, the mixture turned into a gel-like suspension after chitosan was added into a Hoagland base liquid. Based on experimental measurements, 1% w/v chitosan is 13.72 times more viscous than water [31] with an osmolality of approximately 94 mOsm/kg in Hoagland solution. The osmolality is calculated from 72 mOsm/kg of chitosan in pure water [31], plus values from other salt ingredients [32]. Typical osmolality in plant cells ranges from 300–700 mOsm/kg [33], hence an increase in osmolality caused by chitosan should have minimal impact on plant nutrient uptake and cellular tonicity. Whereas, increase in viscosity could conceivably contribute to a creation of physical barrier that could limit root growth. However, previous studies have demonstrated that root growth is not impeded by 1% agar solutions. Viscosity of 0.8–1% agar which is normally supplied into plant growth media is approximately 30–80 times higher than water (depending on pH and temperature) [34]. As shown in several morphological studies, plant roots can penetrate through agar media and demonstrate natural growth [30, 35]. Therefore, the reduction of root expansion is likely a direct effect of chitosan on the plant root system.

**Fig. 5:**
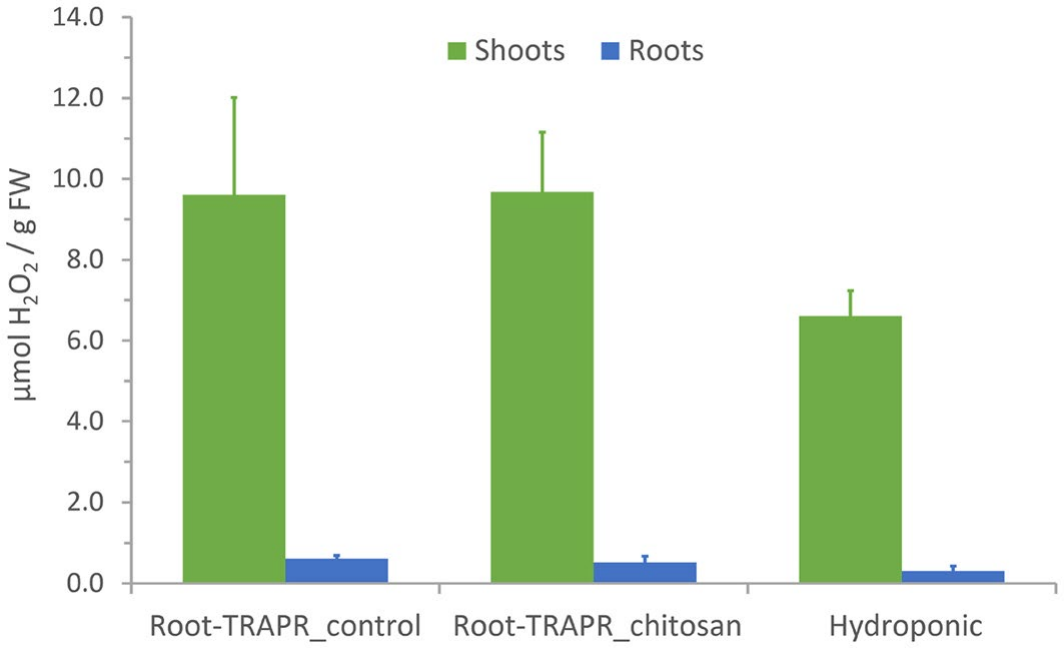
Hydrogen peroxide contents measured from shoots and roots of control and chitosan-treated plants grown in the Root-TRAPR systems compared to the plants grown in standard hydroponic solution. Values are an average of three biological replicates displaying SEM in the error bar. Statistical one-way ANOVA with Tukey's HSD test was used to compare the means across three conditions within the same tissue types, but statistically significant differences were not observed

To assess the health of the plants grown in the Root-TRAPR system, hydrogen peroxide (H_2_O_2_) content was measured in root and shoot tissues and compared to the plants grown in a mini hydroponic-like system (Additional file 2). H_2_O_2_ is a key signaling molecule in plant response upon environmental stresses. Increase in H_2_O_2_ level can be caused by natural factors, such as heat, cold, drought and salinity, resulting in redox imbalance and oxidative stress in plant cells [36]. Therefore, cellular H_2_O_2_ level is often used as an indicator for abiotic stress in crop management [37]. In our study, levels of H_2_O_2_ in both shoots and roots were comparable between control and chitosan-treated plants (Fig. 5). In control, it was 9.61 ± 2.40 and 0.61 ± 0.08 µmol/g fresh weight (FW) in shoots and roots, respectively. This was 9.68 ± 1.48 µmol/g FW in shoots and 0.52 ± 0.15 µmol/g FW in roots of chitosan-treated plants. They were slightly higher than those of the plants grown in a hydroponic device (6.61 ± 0.62 and 0.31 ± 0.11 µmol/g FW in shoots and roots, respectively) but statistical analysis using one-way analysis of variance (ANOVA) showed no significant differences for the shoot and root tissues with a p-value of 0.60 and 0.64, respectively. This suggests that plants were not stressed when grown in the Root-TRAPR system and chitosan did not introduce stress. Moreover, H_2_O_2_ content measured from shoot tissues fell within the range detected from the leaves of experimental control plants in other studies. It was 5–10 µmol/g FW in reed [38] and nearly 6 µmol/g FW in marigold [39]. The level was slightly higher than the normal range (0.5–4 µmol/g FW) measured from various plants, including soybean, ground-ivy, bur oak, common blue-violet and red mangrove under natural conditions. However, environmental and experimental factors should be considered [40].

Furthermore, hemp plants grown in the Root-TRAPR systems developed into a smaller size compared to the plants typically potted in soil (Additional file 3). After 3 weeks of propagation, potted plants and control plants in the Root-TRAPR systems produced the same number of leaves (6–10 leaves) and leaf nodes (2–3 nodes). However, plants in the Root-TRAPR system had extremely shorter heights and smaller leaves. Change in size of the plant demonstrated a plant phenotypic plasticity in response to variable environmental growth factors [41]. The Root-TRAPR system could limit root growing area, causing plants to adapt into an unnatural confined space and yielding small plant [42]. In turn, the smaller size of the plant in the Root-TRAPR system would benefit cannabis plant research. It scales down plant size, reducing maintenance costs and saving plant growing space, usually limited in controlled laboratory conditions. As indicated by normal H_2_O_2_ level, the system could be applied to explore molecular mechanisms underlying plant responses to abiotic and biotic stresses, that could then be tested under hydroponic or field conditions. To understand more about physiological properties of the plants grown in the Root-TRAPR system, measuring other physiological parameters, such as chlorophyll content and photosynthesis rate, could be further examined. Nonetheless, the developed Root-TRAPR system meets the needs of a research tool to visualize plant root phenotype and investigate biological changes in plant tissues and exudate upon elicitor challenge.

### Analytical measurements

After completing plant growth observation, shoot and root tissues and root exudate were harvested, and biochemical analyses including enzymatic assays, phytohormone quantification and DNA gene determination were subsequently carried out to demonstrate the utility of the Root-TRAPR system for plant sample collection and the suitability for subsequent assays to examine plant responses. Herein, the results generated from combining analytical techniques were integrated to assess the effect of chitosan treatment on *C. sativa*.

#### Enzymatic activities

Peroxidase and chitinase are well-known plant pathogenesis-related proteins that play an important role in counteracting fungal attacks [43, 44]. This study measured peroxidase and chitinase activities in plant samples of the shoot, root tissues and root exudate. Tissue samples (shoot and root) were harvested on the last day of the experiment (day 14), whereas root exudate was collected twice on day 7 (pre-chitosan treatment) and day 14 (post-chitosan treatment). It was hypothesized that if chitosan treatment could stimulate the production of plant defense enzymes, a corresponding increase of peroxidase and chitinase activities would be observed. In parallel, protein concentrations of the samples were determined using Bradford assay and later used for data normalization.

When normalized to an equivalent amount of fresh tissue weight, total protein pools extracted from shoot tissues were 5–8 times higher than those extracted from the roots (Table 2). However, shoot tissues had lower peroxidase and chitinase enzyme activities than root tissues. In control plants, shoot expressed peroxidase activity at 0.51 ± 0.12 ΔOD/min·mg protein which was approximately 25-fold lower than that detected in root at 13.17 ± 0.80 ΔOD/min·mg protein. In chitosan-treated plants, peroxidase activity in the shoot (0.66 ± 0.11 ΔOD/min·mg protein) was not different from that in control plants but was doubly increased in root tissues (30.80 ± 8.06 ΔOD/min·mg protein) with a marginally significant difference (p-value = 0.09) as relative to control.

**Table 2:** Total protein concentration and enzymatic activities of peroxidase and chitinase from the shoot and root tissues of control and chitosan-treated plants

Plant tissue	Condition	Protein content (mg protein/g fresh weight)	Peroxidase activity (ΔOD/min·mg protein)	Chitinase activity (mmol GlcNAc released/g protein)
Shoot	Control	13.62 ± 1.26	0.51 ± 0.12	0.21 ± 0.02
	Chitosan	10.88 ± 2.96	0.66 ± 0.11	0.21 ± 0.04
Root	Control	2.72 ± 0.33	13.17 ± 0.80	0.51 ± 0.20
	Chitosan	1.27 ± 0.46	30.80 ± 8.06^‡^	0.85 ± 0.11

Values express mean ± SEM from three biological replicates.

^‡^0.05 < p-value < 0.10 (T-test), comparing between control and chitosan conditions

A similar tendency was observed for chitinase activity. In shoot tissues, it was equivalent at 0.21 mmol N-acetylglucosamine (GlcNAc) released per g protein between control and chitosan groups. The activity was slightly higher in root tissues of chitosan-treated plants (0.85 ± 0.11 mmol GlcNAc released per g protein) as compared to the control (0.51 ± 0.20 mmol GlcNAc released per g protein) but not significant (p-value = 0.22).

In root exudates (Table 3), measured protein concentrations were correlated with the volume of plant roots—the larger the roots, the higher the number of proteins found in the exudate. In control plants, protein content was measured at 53.80 ± 11.91 µg/ml on day 7 which was approximately tripled by day 14 (146.20 ± 15.52 µg/ml). Proteins in root exudate of chitosan-treated plants were 81.07 ± 16.99 µg/ml on day 7 and only increased to 104.87 ± 11.14 µg/ml by day 14. As observed from root morphology (Fig. 3), chitosan-treated plants barely expanded their root after the treatment (10.98 cm on day 7 and 12.79 cm on day 14 in total root length), which would likely result in decreased protein secreted in its exudate on day 14. Peroxidase activity of the pre-treatment exudate was not different between control (0.04 ± 0.01 ΔOD/min·mg protein) and chitosan groups (0.13 ± 0.04 ΔOD/min·mg protein). However, the activity increased 50-fold to 6.48 ± 2.17 ΔOD/min·mg protein in post-treatment exudate of chitosan-treated plants and was 21.6 times higher than that of control plants (0.30 ± 0.12 ΔOD/min·mg protein) with a p-value of 0.047.

**Table 3:** Total protein concentration and enzymatic activities of peroxidase and chitinase from root exudates of control and chitosan-treated plants

	Condition	Protein content (µg protein/ml retentate)	Peroxidase activity (ΔOD/min·mg protein)	Chitinase activity (mmol GlcNAc released/g protein)
Pre-treatment	Control	53.80 ± 11.91	0.13 ± 0.04	1.67 ± 0.51
	Chitosan	81.07 ± 16.99	0.04 ± 0.01	1.34 ± 0.33
Post-treatment	Control	146.20 ± 15.52	0.30 ± 0.12	0.28 ± 0.02
	Chitosan	104.87 ± 11.14	6.48 ± 2.17*	2.02 ± 0.89

Values express mean ± SEM from three biological replicates.

*p-value < 0.05 (T-test), comparing between control and chitosan conditions

The result was similar for chitinase activity. Before the treatment, chitinase activity of pre-treatment exudates was relatively comparable between control (1.67 ± 0.51 mmol GlcNAc released per g protein) and chitosan groups (1.34 ± 0.33 mmol GlcNAc released per g protein). After the treatment, the activity increased to 2.02 ± 0.89 mmol GlcNAc released per g protein in the chitosan group but dropped to 0.28 ± 0.02 mmol GlcNAc released per g protein in control. The difference was approximately 7.2 times, but the statistical test (T-test) showed no significant difference with a p-value of 0.12 between these comparisons due to high variation among the three replicates of the chitosan group, which displayed a coefficient of variation (CV) = 76.13%.

All enzymatic activity data detected from shoot and root tissues and pre- and post-exudate of each assay were combined and plotted together in the same graph (Additional file 4), featuring an overview result of bioactivities. After being treated with colloidal chitosan for 7 days, peroxidase and chitinase activities measured from the root of chitosan-treated plants were 2.3 and 1.7 times higher than those of control plants. The differences were much greater in root exudates as peroxidase and chitinase activities were 21.6 and 7.2 times higher in the chitosan group. This implies that plants produce more peroxidase and chitinase enzymes in root tissues and secrete them into the exudate in response to chitosan treatment. By contrast, peroxidase and chitinase activities were not different between the shoots of control and chitosan-treated plants. This could be because chitosan had a localized impact on protein expression where plant roots were directly exposed to chitosan, but the effect did not transfer to aboveground tissues.

#### Phytohormone content

Phytohormones play a crucial role in plant defense from biotic stresses caused by living organisms, including herbivores, insects, bacteria, fungi and viruses [45]. Salicylic acid (SA), jasmonic acid (JA) and ethylene (ET) are on the front line of combat, functioning as signal molecules once a plant detects pests and pathogens. Other hormones such as abscisic acid (ABA), auxins, cytokinins, gibberellins and brassinosteroids, typically associated with plant growth and abiotic stresses, are induced later through SA, JA and ET signaling networks [46]. This study aimed to verify whether challenging industrial hemp with exogenous chitosan modulates the production of plant hormones. Using a liquid chromatography-mass spectrometry (LC–MS) machine coupled with multiple reaction monitoring (MRM) detection mode, a targeted metabolomics approach was employed to quantify phytohormones levels in shoot and root tissues. Zeatin, indole-3-acetic acid (IAA) and brassinolide (BL) were detected as representatives of cytokinin, auxin and brassinosteroid phytohormone classes, respectively. In addition to major hormones, JA derivatives, jasmonyl-isoleucine (JA-Ile) and 12-oxo-phytodienoic acid (OPDA) and cinnamic acid (CA), a growth-stimulating compound [47] were also analyzed.

Phytohormone levels varied in a wide range from 14.10 ± 0.79 ng of IAA to 35.76 ± 12.62 mg of OPDA per 1 g tissue FW. Zeatin and BL were undetectable in all samples. All quantifiable phytohormones were higher in shoots (Table 4 and Additional file 5). For example, JA and JA-Ile were only observed in plant shoots. SA content in the shoot of control plants (5888.00 ± 2416.20 ng/g FW) was approximately 250 times higher than in the roots (23.85 ± 8.92 ng/g FW). When comparing overall phytohormone content between control and chitosan conditions using principal component analysis (PCA), shoot samples of both groups were separated in the score plot of the first two components (Additional file 6). This indicates that treating industrial hemp with chitosan affects phytohormone production. An approximately tenfold difference was found between the control shoots (5888.00 ± 2416.20 ng/g FW) and chitosan-treated plants (608.53 ± 39.42 ng/g FW) for SA. Whereas, JA, JA-Ile, OPDA, and CA levels were approximately 2–6 times higher in control plants. The observed lower concentrations of phytohormones in shoot tissues of chitosan-treated plants might be due to plant adaptation influenced by root growth disruption. As noted from morphological data, root development was impeded after chitosan was introduced on day 7. Although decreased growth was not yet observed in shoot tissues, adaptive responses are likely to start sharing between the above- and below-ground plant tissues.

**Table 4:** Phytohormone contents detected from shoot and root tissues of control and chitosan-treated plants

	Phytohormone contents (ng/g fresh weight)					
	Control			Chitosan		
	Shoot	Root	Root/shoot ratio	Shoot	Root	Root/shoot ratio
SA	5888.00 ± 2416.20	23.85 ± 8.92	0.004 ± 0.001	608.53 ± 39.42	18.73 ± 2.76	0.030 ± 0.003*
JA	205.67 ± 92.80	N.D	N.D	34.56 ± 1.55	N.D	N.D
JA-Ile	272.50 ± 116.29	N.D	N.D	51.09 ± 11.65	N.D	N.D
OPDA	35,758.87 ± 12,622.87	8552.14 ± 2732.78	0.26 ± 0.02	15,586.85 ± 3799.29	13,053.10 ± 4101.16	0.79 ± 0.07*
ABA	716.94 ± 129.09	96.62 ± 4.69	0.15 ± 0.03	478.62 ± 85.24	170.55 ± 6.82*	0.41 ± 0.10*
IAA	21.70 ± 1.25	14.43 ± 1.46	0.67 ± 0.08	14.06 ± 0.79*	16.82 ± 1.52	1.22 ± 0.17*
CA	259.48 ± 23.90	47.73 ± 8.48	0.16 ± 0.03	44.88 ± 4.61*	55.28 ± 2.39	1.19 ± 0.13*
Zeatin	N.D	N.D	N.D	N.D	N.D	N.D
BL	N.D	N.D	N.D	N.D	N.D	N.D

Values express mean ± SEM from three biological replicates.

*N.D *not detectable

*p-value < 0.05 (T-Test), comparing between control and chitosan conditions within the same type of plant tissues

The levels of phytohormones were comparable in the root tissues between control and chitosan-treated plants (Table 4 and Additional file 5). The PCA plot did not clearly distinguish between both groups (Additional file 6). For instance, SA contents were 23.85 ± 8.92 and 18.73 ± 2.76 ng/g FW for control and chitosan-treated plants, respectively. OPDA, IAA, and CA contents were slightly higher in chitosan conditions. However, the concentration of ABA was significantly higher in the chitosan group (170.55 ± 6.82 ng/g FW) relative to the control (96.62 ± 4.69 ng/g FW) with a p-value of 0.0001. The increasing amount of ABA under chitosan conditions would be correlated to lesser root development in chitosan-treated plants. ABA has been reported to inhibit lateral root formation in *A. thaliana*, pea and tomato [48, 49]. However, the overall effect of ABA on plant root formation is diverse depending on ABA concentration, plant age and environmental factors [50]. ABA also interacts with auxin and ethylene signaling pathways in controlling plant root growth [51], so it is difficult to predict a direct effect of ABA in the root system [52]. Conversely, unchanged levels of other hormones would be a consequence of the bilateral effect of chitosan on root tissues. Cellular phytohormones might be produced less because of reduced root development. On the contrary, the plants might modulate defense signaling pathways in response to chitosan recognition, leading to a rebound of overall phytohormone contents in the root system. However, this is an early assumption and requires further investigation to confirm the effect of chitosan on phytohormone contents in root tissues.

The difference in phytohormone levels between control and chitosan-treated plants was more evident when comparing the proportions between roots and shoots (Table 4). Root-to-shoot ratios of all detected phytohormones were significantly higher in the chitosan condition than the control. This result pinpoints that plants respond towards exogenous chitosan treatment through phytohormone production.

#### Nucleic acid extraction and gene detection

Plant defense-related genes such as chitinases are main targets for determining gene expression in response to chitosan treatment [53, 54]. These genes have not been characterized but only computationally predicted from the *C. sativa* draft genome [55]. The genome was constructed from medicinal cannabis Purple Kush, industrial hemp Finola, and USO-31 varieties. We conducted a simple DNA-PCR experiment to confirm the presence of selected defense genes and to demonstrate that nucleic acids can be readily isolated on plant samples collected from the Root-TRAPR system, alongside metabolite and protein analyses as shown in previous sections. It was also carried out to ensure that chitosan does not interfere with nucleic acid extraction steps. DNA was extracted from the shoot and root tissues of control and chitosan-treated plants. Primers of all genes were designed based on the *C. sativa* draft genome using the NCBI primer-BLAST tool [56]. Actin, ubiquitin and elongation factor-1 alpha (EF-1α) were selected as reference genes. Three chitinase isoforms, chitinase 5, chitinase 2 and chitinase 4-like were selected as primary targets for monitoring DNA analysis. Gene information and primer details are supplied in Additional file 7. Traditional PCR method coupled with ethidium bromide staining was used to analyze DNA amplification products.

**Fig. 6:**
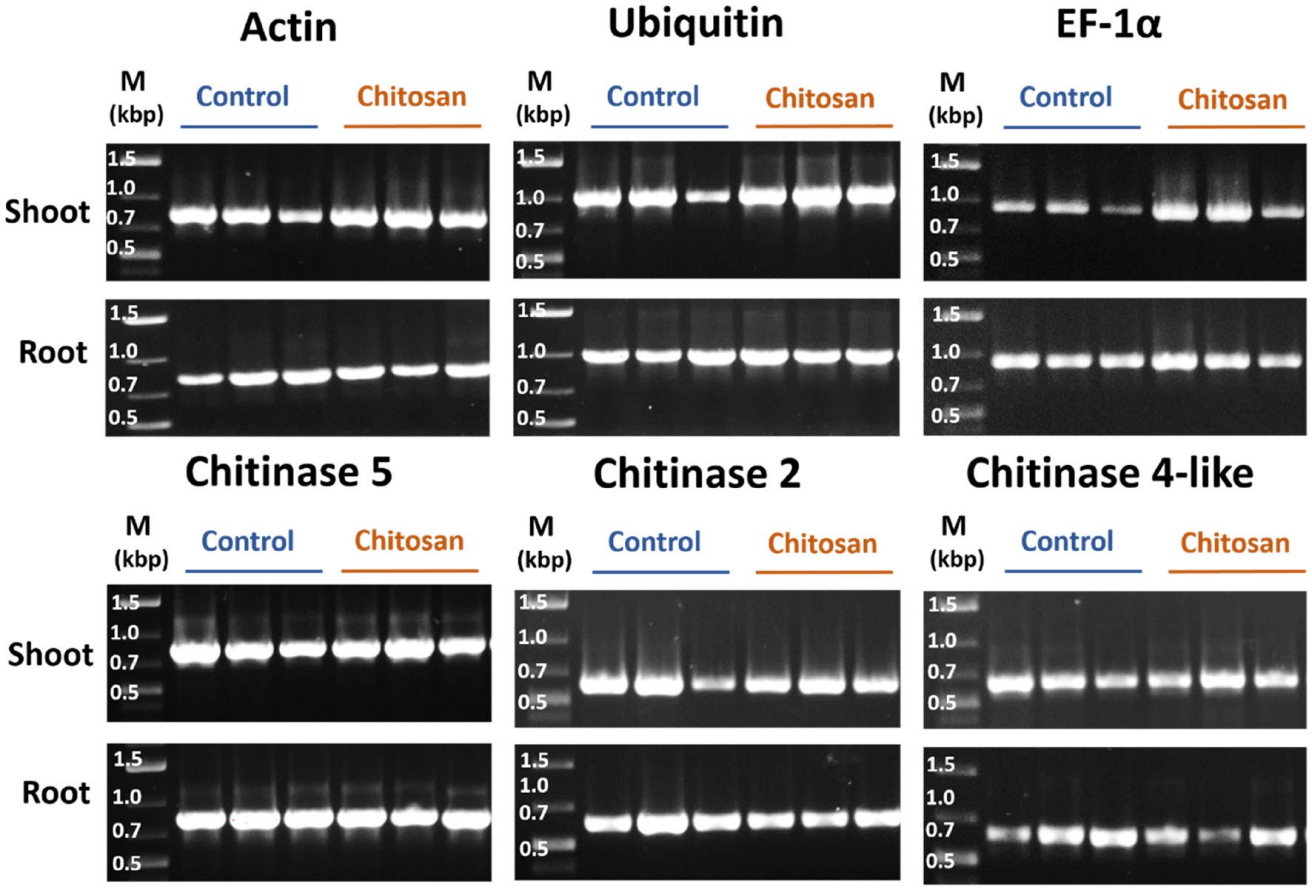
Gel images showing endpoint PCR products of six *C. sativa* genes including actin, ubiquitin, EF-1α, chitinase 5, chitinase 2 and chitinase 4-like comparing between three biological replicates of control and chitosan-treated plants in both shoot and root tissues. M is a DNA marker (GeneRuler 1 kb plus, Thermo Scientific) with DNA sizes in kilo base pair (kbp) unit

Before undertaking PCR reactions, DNA from all samples was measured and normalized to 100 ng/µl. As shown in Fig. 6, amplification and detection of all target genes was achieved in both shoot and root samples. In root tissues, amplicons of actin, ubiquitin and EF-1α genes were relatively comparable between control and chitosan-treated plants. In shoot tissues, the third control replicate showed slightly lesser DNA copies than the first two replicates for all genes. PCR bands of EF-1α detected from the shoots of chitosan-treated plants were slightly denser than those of controls, suggesting EF-1α would not be an ideal reference gene for quantitative analysis. The levels of chitinase 5, chitinase 2 and chitinase 4-like genes were relatively comparable between control and chitosan-treated plants in both shoot and root tissues (Fig. 6). Since chitosan has been reported to bind with nuclear chromatin and damage DNA structure in pea [57], these PCR results showed that chitosan does not impair the process of nucleic acid extraction or amplification.

Future directions for gene analysis include implementing quantitative PCR methods such as real-time and digital PCR to investigate gene expressions at the transcription level (RNA) to explore the molecular effects of chitosan. In addition, other genes involved in the plant defense system, for instance, mitogen-activated protein kinase (MAP kinase), catalase and glucanase, will be included to extend the understanding of chitosan effect on plant defense-related genes.

## Discussion

### Root-TRAPR system—a new plant cultivation device based on the EcoFAB model

In this study, we have developed the Root-TRAPR system, a growth device that is large, modular, reusable, and easy to fabricate. The system is designed to accommodate the largest microscope slide available (128 × 85 mm) and to suit industrial hemp, an economic crop that grows to a larger size than model plants. Compared to earlier growth devices, including the EcoFAB model [9], our system is fabricated from different materials and manufacture techniques, which offers an option for users interested in monitoring plant root growth to build or modify the models. A significant advantage of the Root-TRAPR system is that the system is modular and does not require a plasma cleaner to bond the microscope slide with a cast PDMS root growth chamber. Additionally, the internal root growth chamber can be easily created and modified with our design that uses a PDMS gasket sandwiched between a bottom microscope slide and a top acrylic sheet. These components are enclosed within a 3D-printed PLA frame, secured with nylon bolts and nuts. The PLA frame, stand, and window shutter can be easily manufactured using any 3D printer. The design files are provided in Additional files 8, 9, 10, 11, 12, 13 in a standard STL file format. Materials including acrylic sheet, nylon screw and rubber stopper are accessible from general hardware stores. In addition, the assembled model can be simply cleaned using alcoholic solvents and reused multiple times to reduce unnecessary lab waste generation.

Furthermore, printing materials can be substituted with other types of 3D printing plastic, such as polyetherimide (PEI) and polyether ether ketone (PEEK), which are highly robust, temperature-insensitive and chemically inert [58, 59]. However, these materials are significantly more expensive. The PDMS gasket, which we manually cast in a 3D-printed mold, could also be created using advanced 3D printing technology such as PolyJet printer, which is compatible with a wide range of resin materials, including rubber-like and polypropylene-like photopolymers [60]. Furthermore, the modular nature of the Root-TRAPR system allows for different sized gaskets, which enables fine-tuning of the growth chamber volume by increasing or decreasing the oval gasket diameters or the gasket thickness. This can be achieved by redesigning specific molds or directly printing different gasket sizes. Glass microscope slides can be replaced by an identical-sized plastic acrylic sheet in scenarios where examining root microanatomy or microbe localization using microscopes is not required for instrumental analysis. In addition, other types of transparent plastic, such as polycarbonate (PC) sheets, can be used to replace any top or bottom layer.

The results show that the root morphology of the plants grown in the Root-TRAPR system can be directly observed using a root scanner (Fig. 3). Collection of root exudates in small volumes is achievable and can be promptly processed to extract proteins in a single step of centrifugation. The plant shoot and underground parts can be readily collected from the model and subjected to instrumental analyses for examining plant biochemical responses. The Root-TRAPR system can be applied to a range of other plant physiological studies, such as elucidating plant nutrient uptake mechanisms, investigating plant stress responses in different environments, and exploring plant-microbe interactions occurring underground. However, the system requires specific environmental conditions to maintain sterile conditions before microbial observations. When grown in the current Root-TRAPR system, plants are exposed to an open space and could be contaminated by surrounding microorganisms, interfering with the experiment. To establish a closed system, experiments may be conducted in dedicated growth chambers or within a light transparent sterile box to house the whole plant-inside-Root-TRAPR unit. Sterile conditions will also facilitate further investigation of mechanisms underlying fungal infections in *C. sativa* plants and the effect of chitosan treatment after the plants are already affected by pathogens.

### Chitosan effect on industrial hemp

Chitosan is a well-known plant biostimulant used to stimulate plant growth, induce plant abiotic stress tolerance, and enhance plant pathogen resistance [24, 25]. The compound has been recorded to benefit various crops in different ways [61]. For example, seed soaking in chitosan solution before sowing can increase the production of antioxidant compounds in sweet basil [62]. In tomato, foliar spraying with chitosan can promote crop yield and reduce the severity of bacterial and fungal infections [63]. Post-harvest coating of mango fruits with edible chitosan can prolong shelf-life by delaying the progression of microbial diseases [64]. However, mechanisms underlying the beneficial effects of chitosan have remained unclear [65]. Although chitin, an acetylated form of chitosan, can bind to specific receptors such as chitin elicitor receptor kinases (CERKs) and chitin elicitor binding proteins (CEBiPs) on plant cell membranes to trigger overall plant immunity [66], chitosan has a low affinity to those receptors and does not induce any signaling cascades in plant immune pathways [67, 68]. Based on current understanding, challenging plants with chitosan enhances cellular levels of secondary messengers, H_2_O_2_ and nitric oxide (NO), and phytohormones including ABA, JA and SA, and eventually manipulates expression of defense-related genes inside the nucleus [69]. Changes may lead to increased production of antioxidant compounds and catalytic enzymes such as catalase, chitinase, peroxidase, superoxide dismutase, and phenylalanine ammonia-lyase [61]. Besides enhancing cellular defense mechanisms, chitosan can also induce the secretion of plant defense molecules, including phytohormones and phenolic acids, into extracellular space or exudate [70].

Our findings on industrial hemp are consistent with the benefit of chitosan on plant defense. This study monitored two significant plant defense enzymes, chitinase and peroxidase, and phytohormones. After treating plant roots with 1% w/v colloidal chitosan for a week, peroxidase and chitinase activities increased in root tissues and exudate (Tables 2 and 3). The increases were more intense in root exudate, which was 21- and 7-times higher than the control. In contrast, root tissues showed only approximately 2-times increases for both enzymes. Enzymes in roots induced by chitosan may be secreted into the rhizosphere to add another layer of protection for the plant. However, peroxidase and chitinase activities remained unchanged in the shoots. This suggests that chitosan may affect plant biology at local exposure sites as only the root tissue was directly exposed to chitosan. The effect was not transferable to other distant parts of the plant, as the shoot showed no difference in enzymatic activities.

Furthermore, chitosan reduced phytohormone levels in shoot tissues but did not change the levels in root system (Table 4). Lower levels of hormones in the shoots would correlate to plant growth, which tended to slow down after exposure to a high chitosan concentration. The observation of increased levels of ABA in root tissues of chitosan-treated plants appears correlated to an underproduction of branch roots [48]. The root-to-shoot ratio of all phytohormones was higher in the chitosan condition. This might be because of an inducing effect of chitosan to promote the production or accumulation of plant defense hormones in the root system. Increased plant defense hormones such as SA and OPDA could crosstalk with other hormonal signaling pathways [71], thereby activating the biosynthesis of other hormones and balancing their levels in the root cells. Nonetheless, this is an early observation from the quantitative data and requires further exploration and additional perspectives from proteomics or transcriptomics to verify the outcome comprehensively.

Gene detection was also conducted in this study to initiate PCR methods for future gene expression analysis on plant defense-related genes. Three chitinase genes, including chitinase 5, chitinase 2 and chitinase 4-like, were successfully amplified using the primers designed from the *C. sativa* draft genome sequence [55]. In addition, potential reference genes including actin, ubiquitin and EF-1α were all detectable. This result indicates the reliability of the draft genome to be used as a template for designing primers and studying gene analysis on different *C. sativa* cultivars.

**Fig. 7:**
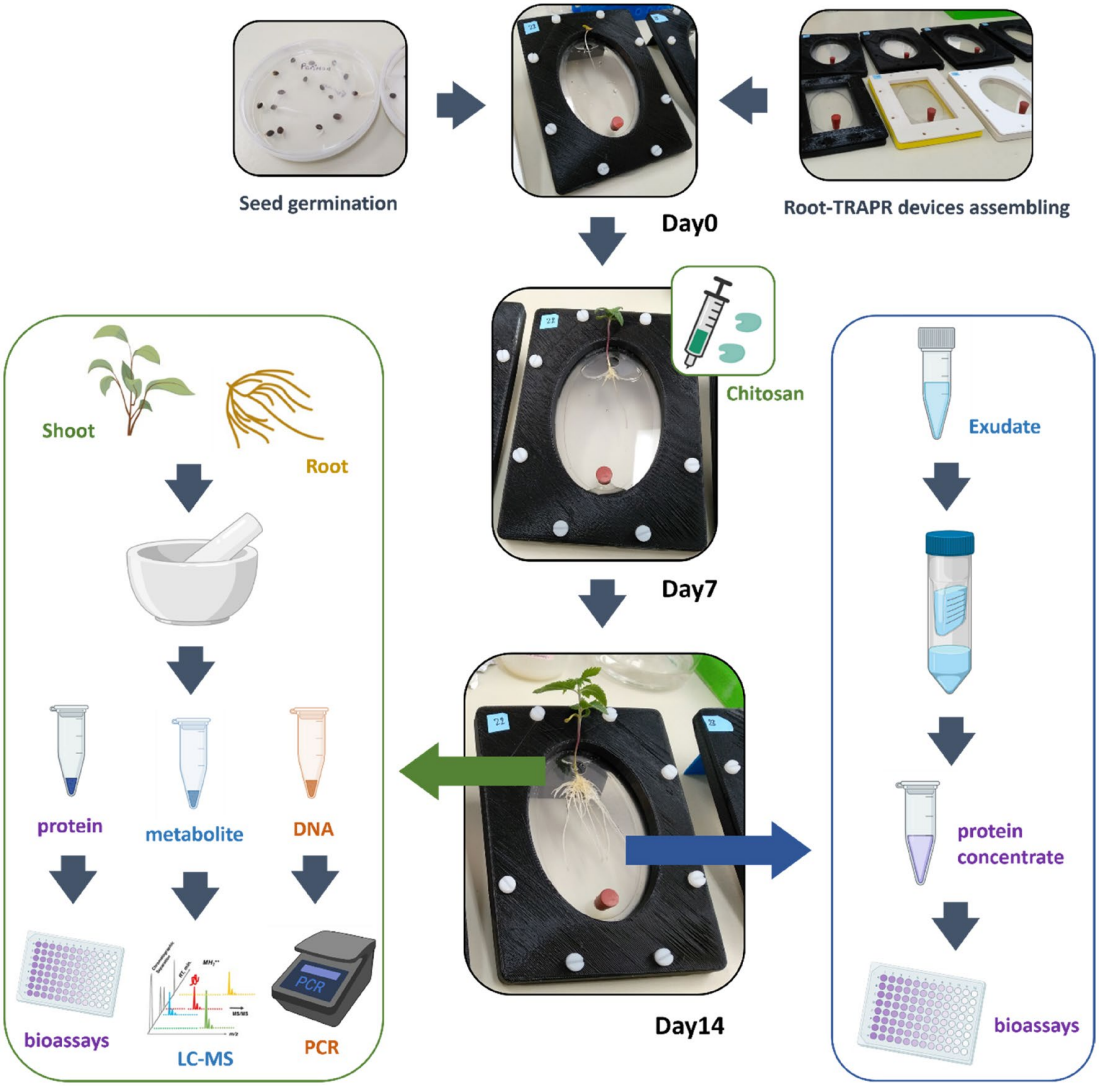
Summarized workflow of plant growth experiment in this study. First, industrial hemp seeds were germinated in Petri dishes, while Root-TRAPR systems were assembled. Seedlings were transferred to Root-TRAPR systems on day 0. Chitosan treatment was performed on day 7. Plant tissues (shoot and root) and root exudate were sampled on day 14. Tissue samples were ground and divided into three parts for subsequent analyses. They were analyzed for bioassays (protein), phytohormone quantification (metabolite) and gene detection (DNA). Root exudate was passed through a 10 kDa MWCO Amicon centrifugal filter device (Merck Millipore, Germany) to concentrate proteins, then tested for enzyme activities. This figure was partially created with BioRender.com

This research has also optimized and established an experimental workflow for exploring biological responses of *C. sativa* towards exogenous stimuli (Fig. 7). The protocol is straightforward but comprehensive, combining three analytical approaches, including genomics, metabolomics and enzymatic assays, to understand plant responses from different perspectives. The workflow is not only limited to studying plants grown in the Root-TRAPR system but applicable for use with plants grown in the original EcoFAB device, typical hydroponic setup or other plant-growing systems. Other plant pathogenesis-related proteins, for example, catalases, glucanases, superoxide dismutases and thaumatin-like proteins, should be included in further studies to add further depth to the current findings.

Furthermore, experiments in this study were conducted using three biological replicates to demonstrate and test the reproducibility of the Root-TRAPR system as an alternative platform for growing *C. sativa* plants and collecting samples to perform analytical measurements. The results demonstrate that the samples harvested from the Root-TRAPR systems can be successfully used for plant physiological and biological analyses, meeting our primary objective. However, higher replication of plant numbers or optimizing seed germination protocol to make growth from seeds more consistent should be considered for future experiments to address the high variation observed in this study. The observed variation is likely to reflect the inherent biological variability of industrial hemp seeds, Ferimon variety, used in this study. Ferimon is a cropping seed-type cultivar and not generally used for research purposes.

Finally, the effect of chitosan has not been reported on *C. sativa* in either industrial hemp or medicinal cannabis cultivars. This is the first study showing that chitosan can potentially trigger the defense system of cannabis plants. Applying to the fields could benefit agriculture in both industrial hemp and medicinal cannabis sectors because they are affected by the same pests and pathogens. However, before being introduced into a disease management scheme, the result must be verified in large-scale production and actual agricultural sites. The effect of chitosan also varies upon its concentration and formulation. We have found that 1% w/v colloidal chitosan forms a viscous mixture which could affect plant nutrient uptake, water potential, and growth of surrounding microbes. Hence, further study is essential to monitor chitosan effects at lower concentrations to identify the best to preserve chitosan benefits whilst maintaining optimal plant growth. Assessing chitosan effects at later stages of plant development such as budding, flowering, and seed-setting stages would also be worthwhile. In summary, chitosan is an inexpensive resource and readily available from by-products of the seafood industry [72]. It could therefore be a potential elicitor to help counteract fungal diseases in agriculture.

## Conclusion

In this research, the Root-TRAPR system has been developed to grow larger plants under in vitro conditions. The system was tested using an industrial cropping cultivar of *C. sativa*, then exploring the impact of chitosan, a potential defense elicitor molecule, on plants. The system enabled the visualization of root material and the ability to harvest plant tissues and exudates that were then successfully processed for enzymatic activity assays, phytohormone measurements and nucleic acid extraction. After treating the plants with 1% w/v chitosan for 7 days, chitinase and peroxidase activities were promoted in root tissues and exudates. This confirms the effect of chitosan to induce plant defense enzymes associated with increased disease resistance. Finally, the Root-TRAPR system has opened the way for further analysis of *C. sativa* and other larger plants under defined in vitro conditions.

## Methods

### Chemicals

Analytical grade solvents (ethanol, isopropanol, hydrochloric acid, phosphoric acid) were supplied by ChemSupply, Australia. LC–MS grade solvents (acetonitrile and formic acid) were acquired from Thermo Fisher Scientific, US. Deionized water was used during plant growth experiments, whereas Milli-Q water (Merck Millipore, Germany) was used for all analytical processes. Formulation of Hoagland solution is supplied in Additional file 1.

### Root-TRAPR system fabrication

Materials used for fabricating the Root-TRAPR system are listed in Table 1, and the fabrication procedure is described step by step.

#### Printing PDMS mold, external frames and supplementary parts

PDMS mold (Fig. 2e), top and bottom external structural frame (Fig. 2d), stand (Fig. 2h) and window shutter (Fig. 2i) were manufactured using an FDM 3D printer (MakerBot Replicator plus, US). All components were designed using an open-source computer-aided design (CAD) software (FreeCAD, https://www.freecadweb.org/). The design files are supplied in Additional files 8, 9, 10, 11, 12, 13. The infill was set at 80% for the external structural frame and 10% for PDMS mold, stand and window shutter. The objects were printed on a raft base layer with a light fill support underneath. Layer height was set at 0.2 mm with two shells. After printing, the internal surfaces of the PDMS mold were finished with coarse (P80) and fine (P600) sandpapers. Printing scrap and support were removed from all printed items before use.

#### Casting PDMS gasket

A Sylgard 184 elastomer kit (Dow Corning, US) created the PDMS gasket. Standard 10:1 (w/w) ratio of base to catalyst was used. Eighteen g of silicone base was mixed with 1.8 g of curing agent in a disposable foil baking cup. The mixture was placed in a vacuum chamber for 30 min to remove air bubbles and then gently poured into the 3D-printed PDMS mold. Overfill was removed by scraping a ruler across the top surface of the mold. The filled mold was incubated at 55 °C overnight to allow the elastomer to set. The solidified PDMS gasket was slowly cut away from the mold using a single edge razor blade. The completed PDMS gasket is shown in the middle of Fig. 2c.

#### Preparation of the upper viewing window acrylic sheet

An acrylic sheet was cut into the desired size (128 × 85 mm) using a Felder BF-5 combination machine (Felder Group, Austria). Then two circular holes were added using a drill press with appropriately diameter sizes of 8- and 9-mm bits. The upper larger hole (9 mm) was left blank for placing the seed, while the lower smaller hole (8 mm) was firmly stoppered with a rubber bung (Fig. 2g) to stop leakage. A completed acrylic sheet is presented on the left of Fig. 2c.

#### Assembly of Root-TRAPR unit

The completed Root-TRAPR system was assembled by placing the PMDS gasket between a microscope glass slide underneath and an acrylic sheet on top. The three internal components were then positioned inside the pocket of the bottom external frame and enclosed by the top frame. Finally, eight pre-sized nylon bolts and nuts (Fig. 2f) were screwed in to tighten the layers and complete the main assembly. Additionally, during growth experiments, the stand (Fig. 2h) and window shutter (Fig. 2i) can be incorporated to tilt the model at a 25° angle from the ground to promote gravitropism and prevent direct light onto the plant root, respectively.

#### Sterilizing Root-TRAPR system

Before use, the assembled Root-TRAPR system and supporting parts were placed in a plastic container and submerged in 70% ethanol for 30 min and 100% ethanol for another 10 min. It was shaken occasionally to ensure all parts were exposed to the solvent, and the oval root growth chamber was filled throughout. After sterilization, the solvent was drained off, and the model was dried in a laminar flow cabinet. Once seedlings had germinated, the sterilized Root-TRAPR system was rinsed with autoclaved deionized water and filled with 15 ml of full-strength Hoagland solution.

### Colloidal chitosan preparation

Colloidal chitosan was prepared according to the previous method [73] with a slight modification. Five g of chitosan powder (medium molecular weight; Sigma, US) was first mixed with 50 ml of 85% phosphoric acid, followed by slowly adding another 50 ml of the acid with continuous stirring. The mixture was left at 4 °C overnight to form a colloidal suspension. Pre-cooled 500 ml of 50% ethanol was added to dilute the mixture, then left at 4 °C overnight again. The suspension was filtered through Whatman Grade 1 filter paper (Whatman plc, UK), aided by vacuum filtration. Colloidal chitosan was retained in the funnel and then washed with distilled water until pH above 5. The retentate was transferred to 50-ml conical tubes and then lyophilized in an Alpha 1–4 LD plus freeze-drier (Christ, Germany). Before use, dried chitosan was resuspended to 1% w/v in Hoagland solution.

### Seed germination

The overview of experimental workflow starting from seed germination until sample collection is illustrated in Fig. 7. Industrial hemp seeds, Ferimon (France) was received from Southern Hemp Australia. Obtaining and processing industrial hemp (low-THC cannabis) at the University of Melbourne is authorized by Agriculture Victoria, the State Government (authority number 2019/12). The seeds were sterilized with 70% ethanol for 1 min and 0.04% sodium hypochlorite for 10 min, followed by rinsing three times with autoclaved deionized water. Sterile seeds were imbibed at room temperature overnight and transferred to round Petri dishes (90 mm in diameter) containing a moistened filter paper. Germination was conducted in the dark at ambient temperature (approximately 20 °C) for 3 days. Day 0 was counted when the seedlings were transferred into the Root-TRAPR system.

### Plant growth and chitosan treatment

Seedlings with 4–6 cm-long tap root were transferred to the Root-TRAPR systems supplied with 15 ml of Hoagland solution using sterilized forceps. Plants were maintained for 7 days in a CMP6010 growth chamber (Conviron, Canada) at 25 °C for 16 h with light and at 21 °C for 8 h of darkness. Light intensity was set at level 2, and relative humidity was maintained at 60%. The nutrient solution was filled up every 2–3 days to compensate for liquid consumption and evaporation. On day 7, plants were separated into control and chitosan conditions. The entire solution was collected and substituted with a new 15 ml of Hoagland solution in the control group. The pre-treated solution was collected and replaced with 1% w/v colloidal chitosan suspension in the chitosan group. All plants were maintained under the same condition for another 7 days. Hoagland solution (approximately 1–2 ml) was added up every 2–3 days in both groups for liquid compensation.

### Root growth measurement

Root growth was monitored and analyzed under a well-calibrated root scanning system composed of an optical scanner (Epson Perfection V800, Japan) equipped with WinRHIZO Arabidopsis 2019 software (Regent Instruments, Canada). Plant roots were scanned every 2 to 3 days by placing the Root-TRAPR device straight on the scanner's document table without removing the liquid medium, with the scanner lid open. Root measurement was determined in three different parameters – root length, root surface area and average root diameter. The software automatically detected the root region in a greyscale mode. As a result, the roots are visible as brighter regions than the background. A manual adjustment was carried out when the automatic detection misread any root region. The software automatically measured root parameters using standard precision and normal cross detection settings. Pictures showing how the software detected regions and measured root parameters are displayed in Additional file 14. The outermost area labelled in green refers to the analyzed root region, while small inner areas labelled in red are manually excluded from the analysis. The software used different colors to define different root diameters ranging from 0–0.5 mm until 4.5–5 mm. For example, roots in red color refer to root diameter of 0–0.5 mm, and roots in yellow color are 0.5–1 mm in diameter. The summation of root length from all root sizes is the total root length and the root length multiplied by the root diameter is the whole root surface area. Plant shoot and overview plant structure were also photographed using a smartphone camera (Xiaomi Redmi 5, China).

### Plant tissues and root exudate collection

Plant shoot and root tissues were harvested on the last day of observation. Plant shoot included stem and leaves sitting above the node of the cotyledons. Plant root was assigned to all parts developing under the Root-TRAPR root growth chamber. They were ground using mortar and pestle under freezing conditions of liquid nitrogen. Fine tissue powder was separately transferred to three micro-centrifuge tubes in approximate 100 mg by weight (Fig. 7). The tubes were weighed and stored in a -80 °C freezer until further use.

Root exudate was collected twice on day 7 (pre-treatment) and day 14 (post-treatment). First, it was drawn from the Root-TRAPR root growth chamber into a 50-ml conical tube. Next, the solution was spun at 2,500 × g, 4 °C for 20 min to remove debris. Next, the supernatant was transferred to a 10 kDa molecular weight cutoff (MWCO) Amicon Ultra-15 centrifugal filter unit (Merck Millipore, Germany) and then centrifuged at 4,000 × g, 4 °C for 40 min to concentrate root exudate proteins. Approximately 200 µl of protein fraction was captured in the filter unit and stored at -80 °C until further use.

### Biological assays

For tissue samples (shoot and root), 1 ml of 100 mM phosphate buffer, pH 6.5, was added to extract proteins from tissue powder (approximately 100 mg). The tube was vortexed and centrifuged at 13,000 × g for 20 min. The supernatant was collected and stored at -20 °C until assay. For root exudate, concentrate protein (approximately 200 µl) was straightaway assayed as follows.

#### H_2_O_2_ detection

The working solution of titanium tetrachloride (TiCl_4_) was pre-made by slowly adding 100 µl of concentrated TiCl_4_ solution (product code: 208566, Sigma, US) to 100 µl of 6 M hydrochloric acid (HCl) on ice. The mixture was left at 4 °C overnight and then diluted in 10 ml of 6 M HCl.

Twenty µl of tissue extract was mixed with 80 µl of 100 mM phosphate buffer, pH 6.5 in a 96-well microplate. Immediately before detection, 100 µl of working TiCl_4_ solution was added to each well. Absorbance was measured at 415 nm using an EnSpire Multilabel plate reader (PerkinElmer, US). H_2_O_2_ content was calculated against a calibration curve created from serial dilutions of 0.001–0.05% v/v standards.

#### Protein measurement

Bradford reagent (Bio-Rad, US) was diluted five times in deionized water. A 20 µl of protein extract was mixed with 180 µl of diluted Bradford reagent in a 96-well microplate. The mixture was incubated at room temperature for 10 min. Absorbance was detected at 595 nm using the plate reader. Protein concentration was measured against a bovine serum albumin (BSA) standard curve (0–100 µg/ml).

#### Peroxidase activity

Twenty µl of protein extract was mixed with 150 µl of 0.025% H_2_O_2_, diluted in 100 mM phosphate buffer, pH 6.5 in a 96-well microplate. Immediately before the assay, 50 µl of 50 mM guaiacol was added to the solution. Absorbance was measured at 470 nm and repeated every 30 s. The rate of absorbance change on the first 3 min was calculated to represent guaiacol peroxidase activity in a unit of ΔOD/min, normalized to protein amount.

#### Chitinase activity

Dimethylaminobenzaldehyde (DMAB) stock solution was prepared by dissolving 8 g of DMAB pellet in a mixture of 70 ml of glacial acetic acid and 10 ml of 32% HCl. Before the assay, a working DMAB solution was prepared by diluting the stock solution ten times in glacial acetic acid.

Forty µl of protein solution was mixed with 100 µl of 1% w/v of colloidal chitin [74], suspended in 50 mM acetate buffer, pH 5.5 and then incubated at 37 °C for 2 h. The reaction was stopped by centrifugation at 8,000 × g for 10 min. Forty µl of 1 M sodium borate buffer, pH 8.5, was added into a mixture, then incubated at 95 °C for 5 min and cooled on ice for 20 min. Five hundred µl of working DMAB reagent was added into a solution, then incubated at 37 °C for 20 min. The final solution was aliquoted into a 96-well microplate and detected at 585 nm. Chitinase activity was evaluated against GlcNAc standard curve (0.02–2 mM) and expressed as mmole GlcNAc released per 1 g protein.

### Phytohormone measurement

Phytohormones were extracted from tissue powder using 200 µl of 70% methanol supplied with 500 ng/ml of internal standards (d_5_-zeatin, d_2_-IAA, d_7_-CA, d_4_-SA, d_6_-ABA and H_2_JA). Samples were vortexed and centrifuged at 13,000 × g for 20 min. The supernatant was transferred into a glass LC–MS vial containing an insert and injected into a 1200 series LC system equipped with a 6410 Triple Quadrupole MS machine (Agilent, US). Metabolites were separated on Eclipse XDB-C18, 1.8 µm, 2.1 × 100 mm column (Agilent, US). The column temperature was set at 45 °C. Mobile phase A and B were 0.1% formic acid in water and acetonitrile, respectively. The elution gradient was set as follows: 80% A (0–2 min), 80–50% A (2–3 min), 50–5% A (3–12 min), 5% A (12–16 min), 5–80% A (16–17 min) and 80% A (17–23 min). The flow rate was 320 µl/min, and the injection volume was 5 µl. Analytes were ionized using electrospray ionization (ESI) source with capillary voltage at 5500 V and 4500 V for positive and negative modes, respectively. The nebulizer was set at 55 psi. Nitrogen gas flow was maintained at 13 L/min and 250 °C. According to the published method [75], phytohormones were detected using multiple reaction monitoring (MRM) program. The MRM transitions, collision energies and polarities were applied as follows: zeatin (220.1 → 136.1 m*/z*, 14 eV, positive), IAA (176.1 → 130.1 m*/z*, 10 eV, positive), CA (149.1 → 103.1 m*/z*, 20 eV, positive), BL (481.5 → 315.3 m*/z*, 10 eV, positive), SA (137.0 → 93.0 m*/z*, 16 eV, negative), ABA (263.1 → 153.1 m*/z*, 8 eV, negative), JA (209.1 → 59.0 m*/z*, 8 eV, negative), JA-Ile (322.1 → 129.9 m*/z*, 24 eV, negative), OPDA (291.0 → 164.9 m*/z*, 20 eV, negative), d_5_-zeatin (225.2 → 137.1 m*/z*, 20 eV, positive), d_2_-IAA (178.1 → 132.0 m*/z*, 12 eV, positive), d_7_-CA (156.1 → 109.0 m*/z*, 22 eV, positive), d_4_-SA (141.0 → 97.1 m*/z*, 16 eV, negative), d_6_-ABA (269.1 → 159.1 m*/z*, 8 eV, negative) and H_2_JA (211.1 → 59.0 m*/z*, 12 eV, negative). Phytohormone concentrations were measured by comparing relative peak area against calibration curves created from serial dilutions of the standards. The curve was plotted from 4–6 data points in a range of 10–1000 ng/ml according to the phytohormone levels found in the samples.

### DNA extraction and PCR analysis

Four hundred µl of DNA extraction buffer (160 mM Tris, 56 mM EDTA, 30 mM sodium metabisulfite and 1.6 M sodium chloride) was added into tissue powder (approximately 100 mg) and centrifuged at 13,000 × g for 5 min. Three hundred µl of supernatant was taken and mixed with 300 µl of 100% isopropanol. The mixture was incubated at room temperature for 10 min with occasionally tube-inverting and then centrifuged at 13,000 × g for 5 min. The pellet was washed with 300 µl of 70% ethanol and air-dried overnight. The dried DNA pellet was dissolved in 50 µl of nuclease-free water (Qiagen, Germany). DNA concentration was measured using a UV5Nano spectrophotometer (Mettler-Toledo, US).

Six *C. sativa* genes (encoding actin, ubiquitin, EF-1α, chitinase 5, chitinase 2 and chitinase 4-like) were predicted from the *C. sativa* draft genome [55]. Gene and primer details are described in Additional file 7. A 100 ng of DNA template was added to 25 µl of PCR reaction mixture, consisted of 1 × MyTaq Red buffer, 0.5 U MyTaq DNA polymerase (Bioline, US) and 0.4 µM forward and reverse primers each. The PCR amplification was performed using a T100 thermal cycler (Bio-Rad, US) with an initial denaturation of 2 min at 95 °C, followed by 35 cycles of 30 s at 95 °C, 30 s at 55 °C and 1.15 min at 72 °C, and a final extension of 5 min at 72 °C. A 10 µl of the amplification product was resolved in 1% agarose gel electrophoresis at 85 V for 50 min. The gel was stained with ethidium bromide and analyzed using Gel Doc EZ imager equipped with ImageLab software (Bio-Rad, US).

### Statistical analysis

A two-tailed student's T-test was used for enzymatic activity and phytohormone content with Microsoft Excel 2016 software. One-way ANOVA followed by Tukey's honestly significant difference (HSD) analysis was used for root growth measurement and hydrogen peroxide content with Minitab 19 software. A p-value below 0.05 was considered as a significant difference between tested conditions. Online MetaboAnalyst 5.0 software [76] was used to perform principal component analysis (PCA) of overall phytohormone content. Before the analysis, Pareto data scaling was employed to normalize shoot tissue data while the data of root tissue was log-transformed and scaled using mean centering.

(STL files can be opened using FreeCAD software, https://www.freecadweb.org/).

## Supplementary Information

**Additional file 1:** The formula of the Hoagland solution used for growing industrial hemp in this study.

**Additional file 2:** Comparison of industrial hemp after 2 weeks growth in the Root-TRAPR systems compared to a mini hydroponic-like system (50-ml conical tube).

**Additional file 3:** Two-week-old industrial hemp plants grown in potting soil. They grew taller and bigger than the plants grown in the Root-TRAPR systems, but both developed the same number of leaves and nodes.

**Additional file 4:** Summarized protein content, peroxidase and chitinase activities from shoot and root tissues and pre- and post-exudate compared between control and chitosan conditions.

**Additional file 5:** Boxplots showing phytohormone levels in separate graphs compared between control and chitosan conditions.

**Additional file 6:** Principal component analysis (PCA) of phytohormone contents comparing control and chitosan-treated plants in shoot and root tissues.

**Additional file 7:** Gene and primer details of six *C. sativa* genes detected in this study.

**Additional file 8:** 3D design file of PDMS mold.

**Additional file 9:** 3D design file of the bottom external structural frame.

**Additional file 10:** 3D design file of the top external structural frame.

**Additional file 11:** 3D design file of the stand.

**Additional file 12:** 3D design file of left window shutter.

**Additional file 13:** 3D design file of right window shutter.

**Additional file 14:** Pictures showing how WinRHIZO software detects root areas and analyzes root parameters.

## Data Availability

All data generated from this study are included in this published article and supporting materials. Additional details on the Root-TRAPR system fabricating procedures can be acquired from the corresponding author upon reasonable request.

## Abbreviations

3D: Three-dimensional
ABA: Abscisic acid
ANOVA: Analysis of variance
BL: Brassinolide
CA: Cinnamic acid
DMAB: Dimethylaminobenzaldehyde
EcoFAB: Ecosystem fabrication
EDTA: Ethylenediamine-tetra-acetic acid
EF-1α: Elongation factor-1 alpha
FDM: Fused deposition modelling
FW: Fresh weight
GlcNAc: *N*-Acetylglucosamine
H_2_O_2_: Hydrogen peroxide
HCl: Hydrochloric acid
HSD: Honestly significant difference
IAA: Indole-3-acetic acid
JA: Jasmonic acid
JA-Ile: Jasmonyl-isoleucine
LC: Liquid chromatography
MRM: Multiple reaction monitoring
MS: Mass spectrometry
OD: Optical density
OPDA: 12-Oxo-phytodienoic acid
PCA: Principal component analysis
PCR: Polymerase chain reaction
PDMS: Polydimethylsiloxane
PLA: Polylactic acid
Root-TRAPR: Root-transparent reusable affordable three-dimensional printed rhizo-hydroponic
SA: Salicylic acid
SEM: Standard error of the mean
THC: Tetrahydrocannabinol
TiCl_4_: Titanium tetrachloride
