# Supplementary material for: Root-TRAPR: a modular plant growth device to visualize root development and monitor growth parameters, as applied to an elicitor response of Cannabis sativa
Source: Plant Methods. 2022 Apr 9;18:46. doi: 10.1186/s13007-022-00875-1 (PMC8994333; doi:10.1186/s13007-022-00875-1)
Supplement: Supplementary file 1 — Additional file 1: The formula of the Hoagland solution used for growing industrial hemp in this study. [file 13007_2022_875_MOESM1_ESM.docx]

**Additional file 1. Formula of Hoagland solution used for growing industrial hemp in this study**

| **Solution** | **Compound** | **Stock concentration (mM)** | **Volume of stock (ml)**  **per 1 l of final solution** | **Final concentration (µM)** |
| --- | --- | --- | --- | --- |
| **Macronutrients** | | | | |
| 1 | NH_4_NO_3_ | 40 | 5 | 200 |
|  | KNO_3_ | 1000 |  | 5000 |
| 2 | Ca(NO_3_)_2_·4H_2_O | 400 | 5 | 2000 |
| 3 | MgSO_4_·7H_2_O | 400 | 5 | 2000 |
|  | KH_2_PO_4_ | 20 |  | 100 |
| 4 | Fe·EDTA (Na salt) | 50 | 1 | 50 |
| **Micronutrients** | | | | |
| 5 | H_3_BO_3_ | 50 | 1 | 50 |
|  | MnSO_4_·H_2_O | 5 |  | 5 |
|  | ZnSO_4_·7H_2_O | 10 |  | 10 |
|  | CuCl_2_ | 0.5 |  | 0.5 |
|  | MoO_3_ | 0.1 |  | 0.1 |
