# Supplementary figures and images for: Root-TRAPR: a modular plant growth device to visualize root development and monitor growth parameters, as applied to an elicitor response of Cannabis sativa
Source: Plant Methods. 2022 Apr 9;18:46. doi: 10.1186/s13007-022-00875-1 (PMC8994333; doi:10.1186/s13007-022-00875-1)

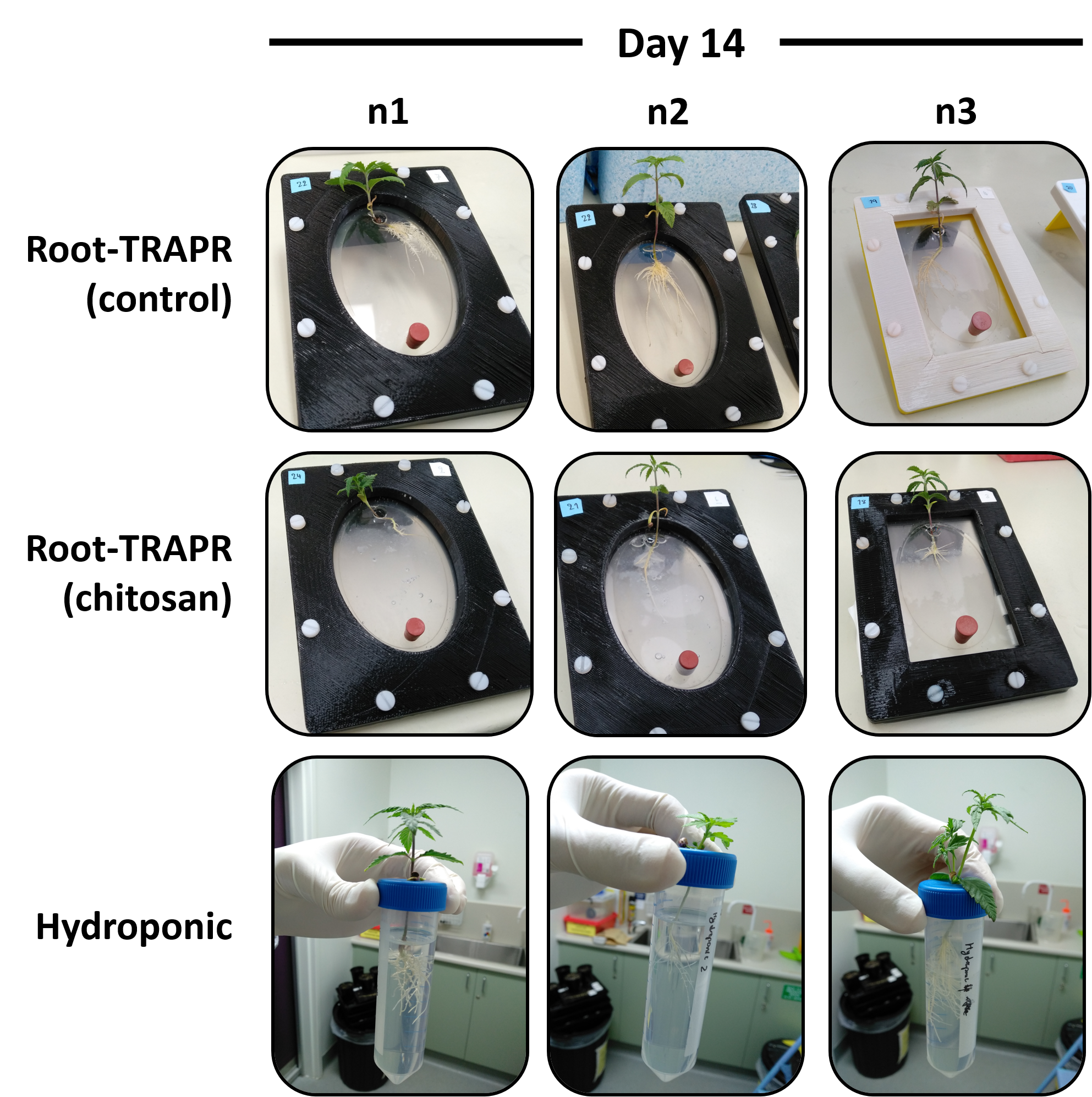

Supplement: Supplementary file 2 — Additional file 2: Comparison of industrial hemp after 2 weeks growth in the Root-TRAPR systems compared to a mini hydroponic-like system (50-ml conical tube). [file 13007_2022_875_MOESM2_ESM.png]

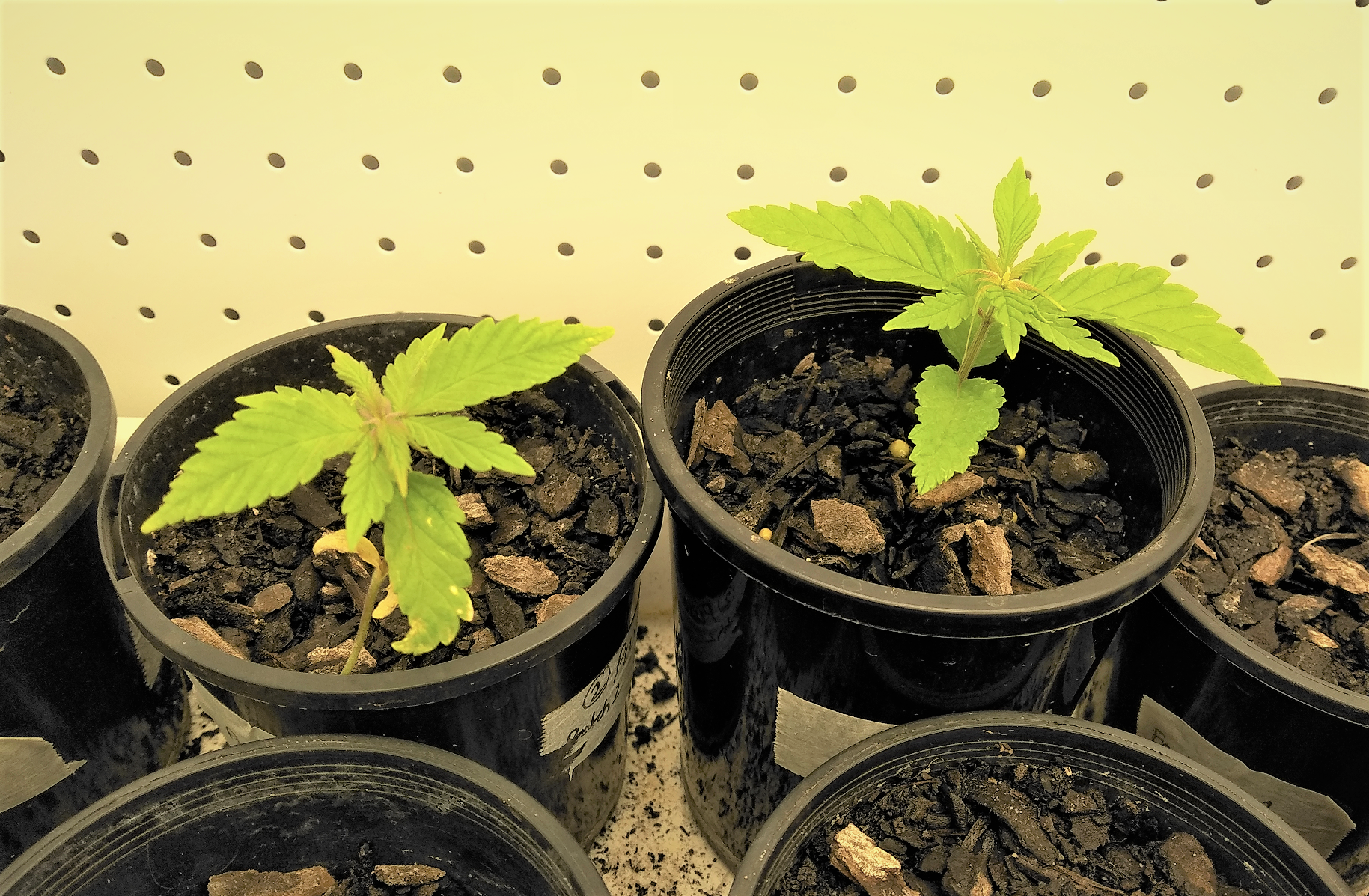

Supplement: Supplementary file 3 — Additional file 3: Two-week-old industrial hemp plants grown in potting soil. They grew taller and bigger than the plants grown in the Root-TRAPR systems, but both developed the same number of leaves and nodes. [file 13007_2022_875_MOESM3_ESM.png]

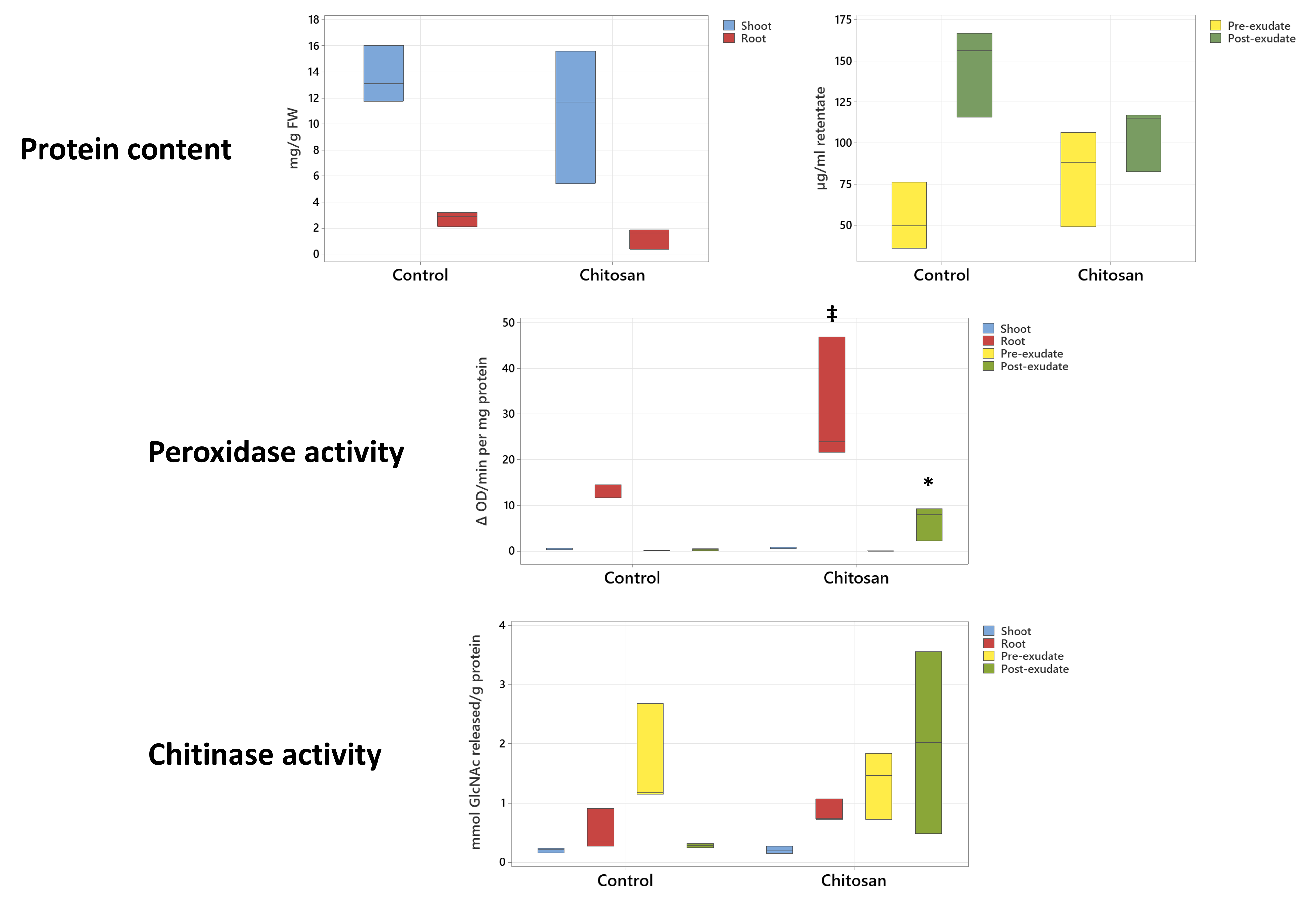

Supplement: Supplementary file 4 — Additional file 4: Summarized protein content, peroxidase and chitinase activities from shoot and root tissues and pre- and post-exudate compared between control and chitosan conditions. [file 13007_2022_875_MOESM4_ESM.png]

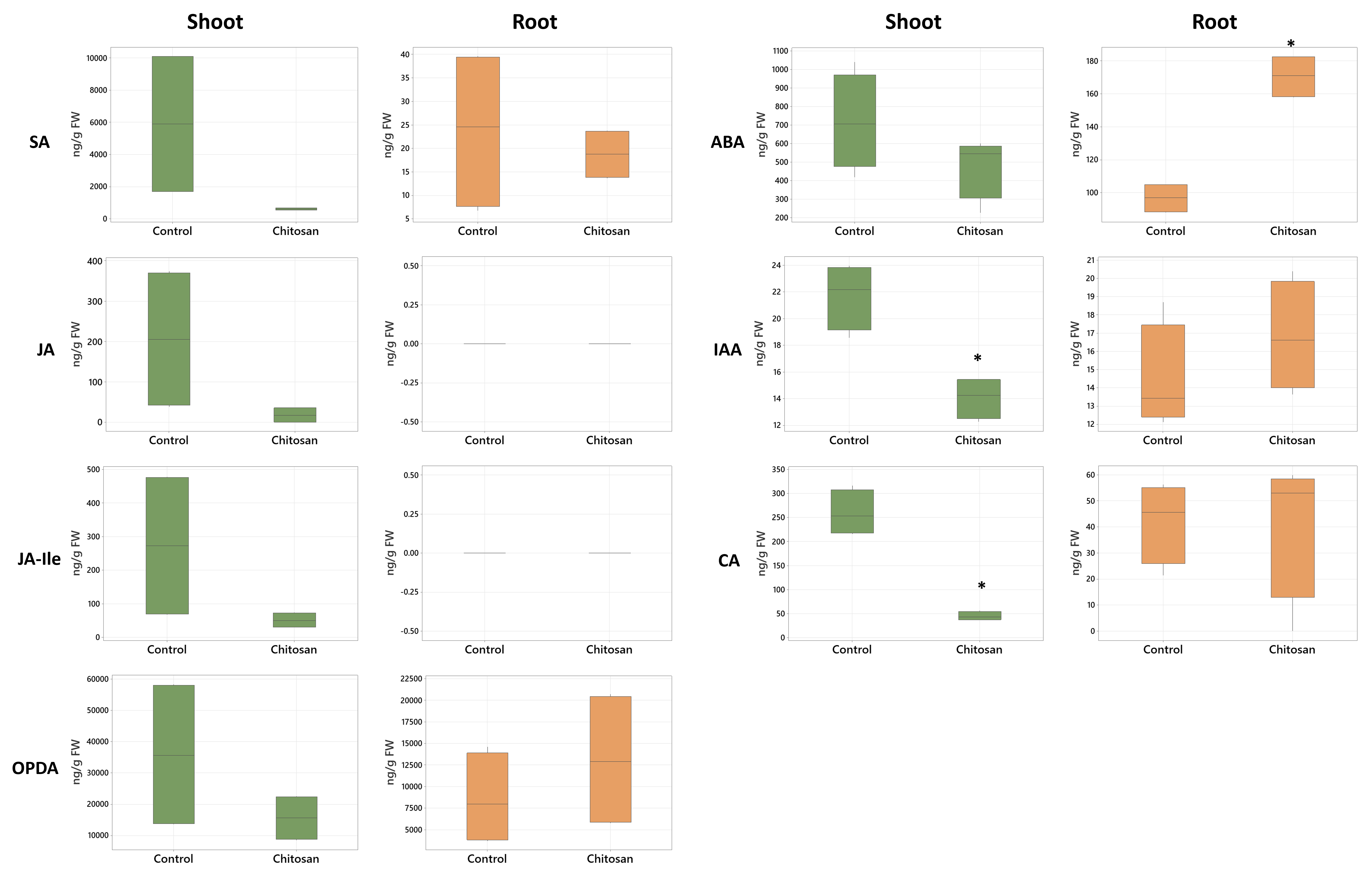

Supplement: Supplementary file 5 — Additional file 5: Boxplots showing phytohormone levels in separate graphs compared between control and chitosan conditions. [file 13007_2022_875_MOESM5_ESM.png]

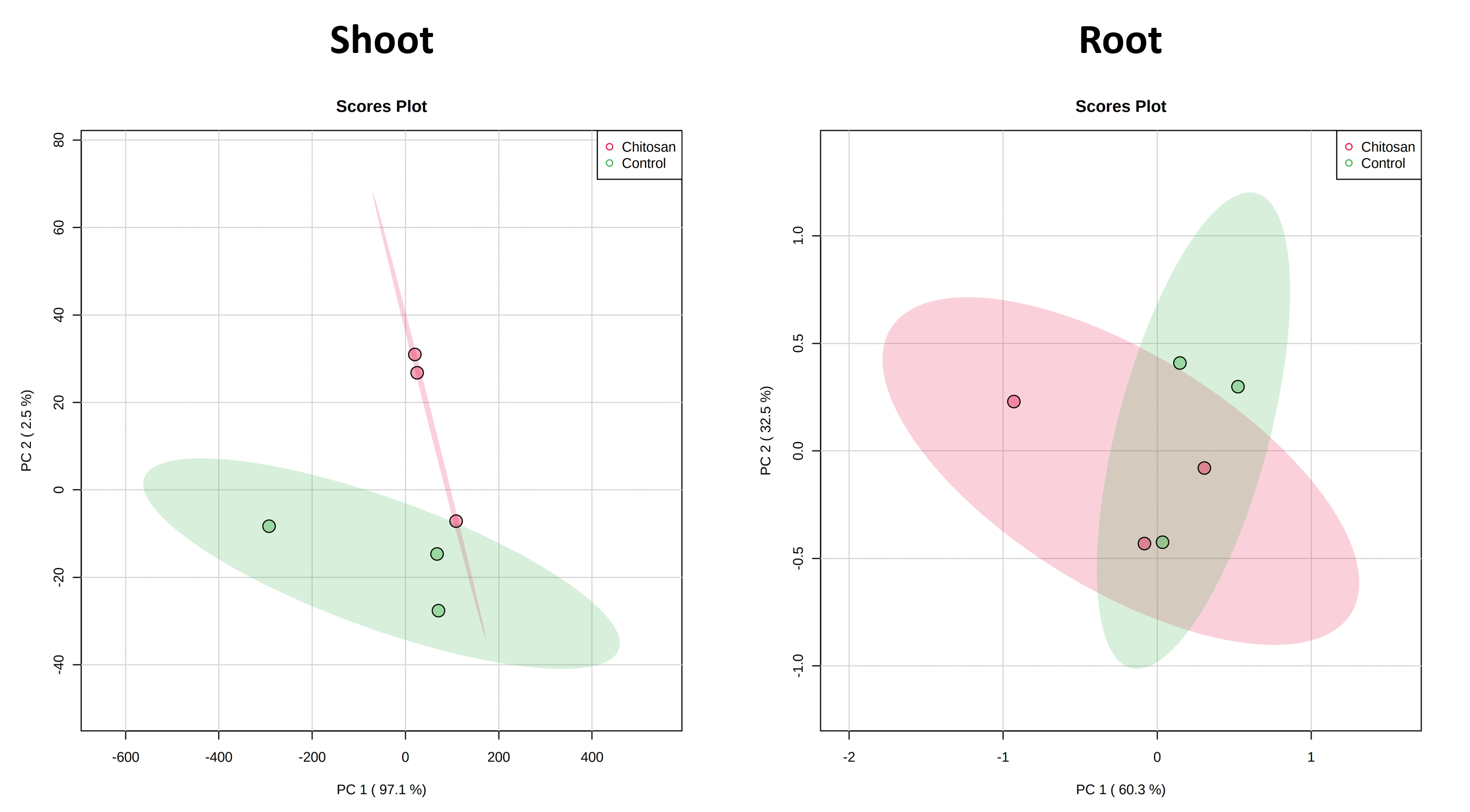

Supplement: Supplementary file 6 — Additional file 6: Principal component analysis (PCA) of phytohormone contents comparing control and chitosan-treated plants in shoot and root tissues. [file 13007_2022_875_MOESM6_ESM.png]

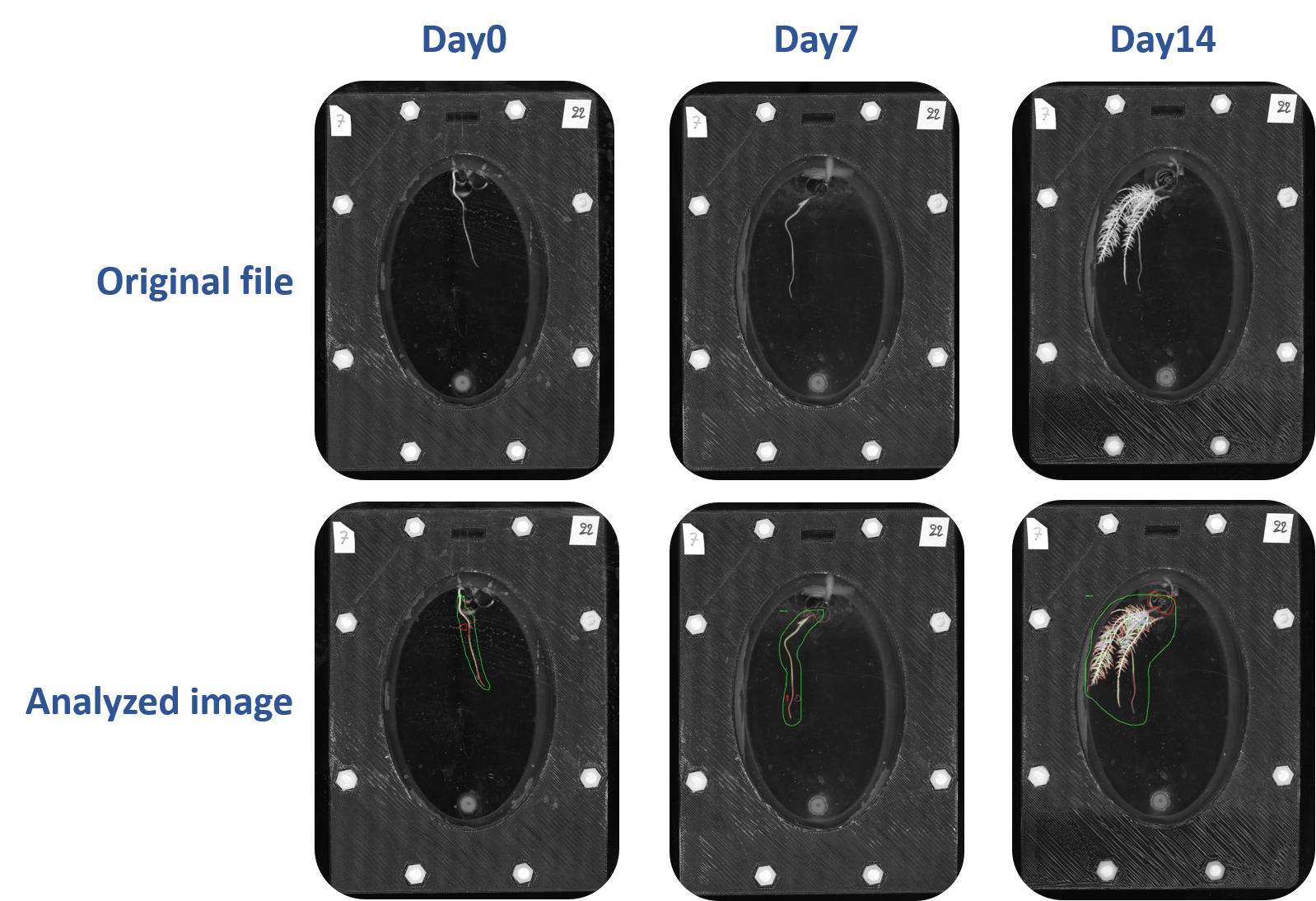

Supplement: Supplementary file 14 — Additional file 14: Pictures showing how WinRHIZO software detects root areas and analyzes root parameters. [file 13007_2022_875_MOESM14_ESM.png]
